# Screening and Preliminary Identification of Inhibin α Subunit-Specific Nanobodies Through High-Throughput Sequencing Combined with Mass Spectrometry

**DOI:** 10.3390/ani16131961

**Published:** 2026-06-25

**Authors:** Jifu Ma, Zhumanov Kairat, Bupebayeva Lyalla, Zhongmei Ma, Wurelihazi Hazihan

**Affiliations:** 1 College of Animal Science and Technology, Shihezi University, Shihezi 832003, China; 2 Department of Microbiology, Virology and Immunology, Faculty of Veterinary Medicine, Kazakh National Agrarian Research University, Almaty 050010, Kazakhstan

**Keywords:** inhibin α, nanobody, FSH

## Abstract

Inhibin, a water-soluble protein emitted by the gonads, plays a pivotal role in regulating the release of follicle-stimulating hormone (FSH) from the pituitary gland, which, in turn, influences follicular growth, gamete production, and the secretion of associated hormones. We performed high-throughput sequencing of the nanobody gene in the lymphocytes of Bactrian camels before and after inhibin α protein immunization followed by mass spectrometry analysis of specific antibodies to this protein in the serum following immunization to screen for inhibin α subunit-specific nanobodies. Seven inhibin α-specific nanobodies, namely Nb-1712, Nb-1971, Nb-2000, Nb-799, Nb-2004, Nb-1737, and Nb-338, were identified through high-throughput sequencing and mass spectrometry. Following the construction and expression of a prokaryotic expression vector, five of these nanobody proteins were successfully produced. These proteins demonstrated high affinity for inhibin α in the indirect enzyme-linked immunosorbent assay. Notably, nanobodies Nb-1737, Nb-1971, and Nb-2004 significantly downregulated *Inha* and upregulated *Fshb* gene expression, enhancing follicle-stimulating hormone secretion. In female mice, these three nanobodies promoted follicular development and led to a numerical increase in litter size (average ~10%, with Nb-2004 showing a 14.93% increase), although the differences were not statistically significant. These findings demonstrate their potential to regulate reproductive function. We identified 7 inhibin α subunit-specific nanobody genes from a Xinjiang Bactrian camel’s lymphocyte genome through high-throughput sequencing and mass spectrometry. We also compared their relative binding affinities and characterized their biological functions, thereby providing key theoretical guidance and technical support for increasing FSH levels.

## Simple Summary

Infertility and low reproductive rates are major challenges in animal farming and human medicine. This study aimed to further validate and expand the use of nanobodies as a potential strategy distinct from conventional hormone therapy. Inhibin normally limits the production of a key hormone called follicle-stimulating hormone, which is needed for egg and sperm development. By neutralizing inhibin, these nanobodies could help the body produce more follicle-stimulating hormone on its own, without the need for repeated hormone injections. The researchers identified seven nanobody candidates from camels using advanced genetic and protein analysis methods. Three of these nanobodies were tested in female and male mice. The results showed that the nanobodies successfully increased follicle-stimulating hormone levels and promoted the growth of egg-containing follicles in the ovaries. Male mice also showed a trend toward better testis development. Although litter sizes increased by about ten percent in treated females, the difference was not statistically significant, possibly because the nanobodies stayed in the body for only a short time. Overall, this work offers a promising, low-cost alternative to traditional hormone therapy and lays the groundwork for future improvements in animal reproduction.

## 1. Introduction

Inhibin is a glycoprotein heterodimer hormone that influences animal reproduction through the hypothalamic–pituitary–gonadal (HPG) axis and is involved in mammalian ovulation [1,2]. The inhibin family mainly consists of inhibin A (INH-A, α-βA heterodimer) and inhibin B (INH-B, α-βB heterodimer), both of which suppress pituitary FSH secretion. Many studies have confirmed that inhibin immunization is beneficial for both male and female mammals. It can increase the serum FSH levels [3], ovulation rate [4,5], and pregnancy rate [4,6,7] of female mammals and increase the sperm count and sperm activity of male mammals [8]. So far, most inhibin-immunity-related studies have involved active immunization. However, active immunization cannot be promoted because of the requirement for a limited number of antigens.

As an alternative strategy to conventional FSH injections for ovarian stimulation, passive immunization with anti-inhibin antibodies neutralizes endogenous inhibin and offers unique advantages: it stimulates endogenous FSH secretion while avoiding ovarian hyperstimulation syndrome and the stress of repeated injections caused by exogenous hormones. Inhibin antibodies are obtained from various sources [9,10,11,12,13,14,15] and are of two types: polyclonal [16] and monoclonal antibodies [17]. Animals immunized with inhibin include sheep [10], goats [16], cattle [13], horses [18], rabbits [11], pigs [12], etc. However, monoclonal antibodies are complex and expensive to produce, while polyclonal antibodies suffer from batch-to-batch variability and lower specificity, affecting their reliability.

The discovery of heavy-chain antibodies, a camelid-specific form of antibodies, has raised high hopes. Camelid sera contain unique types of antibodies composed only of two identical heavy chains and are designated as “heavy-chain-only antibodies” (HCAbs) [19]. The variable domain of HCAb, corresponding to the paratope (also known as the antigen-binding site), is called the variable domain of the heavy chain of heavy-chain antibody (VHH), also known as a nanobody [20,21,22]. Nanobodies have a low molecular weight, strong stability, good solubility, low immunogenicity, and are easily expressed. This solves the problems of high immunogenicity and poor solubility in inhibin antibodies [20,23,24,25]. Furthermore, nanobodies can be efficiently expressed in bacteria at a low cost, making them a potentially economical and feasible alternative to FSH injections for ovarian stimulation. Nanobodies thus allow the possibility for the promotion and use of passive immunization with inhibin. Our previous work successfully selected anti-inhibin-α nanobodies using a phage display library [26]. While that study validated the feasibility of obtaining such nanobodies, it did not comprehensively analyze the full repertoire of VHH sequences induced by immunization.

In this research, we sequenced the total VHH cDNA derived from the lymphocytes of Bactrian camel immunized with the inhibin α subunit (encoded by the *INHA* gene) by using high-throughput sequencing techniques. Subsequently, we performed mass spectrometry to analyze serum anti-inhibin α antibodies, aiming to screen for the inhibin α subunit-specific nanobody gene. Later, the specific nanobody protein was identified through a process including gene synthesis, vector construction, and protein expression. To evaluate the affinity of the selected nanobody for the inhibin α subunit, we performed protein–protein docking simulations and an indirect enzyme-linked immunosorbent assay (ELISA). The biological functions of the top three nanobodies with the highest affinity were elucidated through passive immunization in mice. The present study establishes a foundation for the development of cost-effective and efficient nanobody-based passive immunization strategies against inhibin and offers theoretical insights into the field of animal reproductive immunology.

## 2. Materials and Methods

### 2.1. Animal

A 2-year-old male Xinjiang bimodal Bactrian camel was provided by Huaguoshan Camel Farm in Shihezi City, Xinjiang, China.

### 2.2. Strains and Carriers

*Escherichia coli* BL21(DE3) and DH5α competent cells were purchased from Beijing TransGen Biotechnology Co., Ltd. (Beijing, China). The prokaryotic expression vector of sheep *INHA* (pET32a-*INHA*) was constructed in our laboratory.

### 2.3. Main Reagents

RNA extraction kits, reverse transcription kits, plasmid extraction kits, and gel recovery kits were all purchased from Tiangen Biochemical Technology Co., Ltd. (Beijing, China). The qPCR Mix was purchased from Beijing TransGen Biotech Co., Ltd. (Beijing, China). The camel peripheral blood lymphocyte extraction kit was purchased from Beijing Solarbio Science&Technology Co., Ltd. (Beijing, China). The restriction endonuclease was purchased from Takara Biology Company (Beijing, China). Rabbit anti-camel IgG serum, horseradish peroxidase (HRP)-labeled mouse anti-rabbit IgG, and HRP-labeled mouse anti-camel serum IgG were purchased from Beijing Solarbio Science&Technology Co., Ltd. (Beijing, China). Mouse anti-His tag antibodies and mouse anti-E tag antibodies were purchased from Kingsley Biotechnology Co., Ltd. (Nanjing, China). Primer synthesis and sequencing were completed by Beijing Ruibo Xingke Biotechnology Co., Ltd. (Beijing, China). High-throughput sequencing was performed by Mingke Biotechnology (Hangzhou) Co., Ltd. (Hangzhou, China). The mass spectrometry analysis was conducted by Shanghai Baipu Biotechnology Co., Ltd. (Shanghai, China).

### 2.4. Preparation of Antigen and Immunity of Bactrian Camels

First, total RNA was extracted from the Kazakh sheep ovary tissue, and cDNA was obtained through reverse transcription. The *INHA* gene was obtained through PCR by using the obtained cDNA as the template and *INHA*-*Nde* I-F and *INHA*-*Not* I-R as primers (Table 1, NM_001308579.1). Then, the correctly sequenced *INHA* gene and pET32a plasmid were digested with double enzymes, and the digested products were ligated with DNA ligase following agarose gel electrophoresis. Finally, the ligated products were transformed into *E. coli* BL21 (DE3) cells. The cryopreserved strains were coated on LB (Luria–Bertani) solid medium (containing ampicillin antibiotics). Single clones were selected, monoclonal cultures were completed, and numerous *E. coli*-carrying pET32a-*INHA* vectors were obtained. The recombinant plasmid was confirmed by bacterial liquid PCR, double enzymatic digestion of the plasmid, and subsequent sequencing analysis.

The prokaryotic inhibin α expression strain was cultured in LB liquid medium, and the strain was induced with IPTG. The best induction time and expression form of the inhibin α protein were determined through polyacrylamide gel electrophoresis (no reducing agent was added). The IPTG-induced bacterial solution was centrifuged. The pellet was resuspended with PBS. The pellet was then repeatedly freeze-thawed and ultrasonically disrupted. The products formed through ultrasonic fragmentation were centrifuged to collect the inclusion body (pellet). The inclusion bodies were heavily suspended in solution A (20 mM Tris–HCl, 5 mM imidazole, 0.5 M NaCl, and 8 M urea), dissolved at 4 °C for 16 h, and centrifuged at 10,000 rpm for 30 min. The supernatant was filtered through a 0.45-μm filter and stored at 4 °C. The inhibin α protein was purified through the His-tagged Ni^+^ Agar column. After the purified inhibin α protein was placed into a dialysis bag, it was dialyzed with 6, 4, 2, and 1 M urea and PBS for 6 h. The inclusion body protein was refolded and stored in a refrigerator at −20 °C.

The inhibin α protein was analyzed through polyacrylamide gel electrophoresis. The gel region containing proteins of 30–50 kDa was excised, and the α protein was transferred to the PVDF membrane. The PVDF membrane was blocked at room temperature for 2 h in a 5% skim milk powder solution and cleaned with TBST 3 times, for 10 min each time. The diluted mouse anti-His tag antibody was incubated at room temperature for 1 h and washed with TBST 3 times. The membrane was incubated with HRP-labeled goat antimouse IgG at room temperature for 1 h, and the washing steps were repeated at the end. The enhanced chemiluminescence (ECL) ultra-sensitive detection solution was used to develop the image for 5 min.

Before immunization, non-anticoagulant whole blood (50 mL) and anticoagulant whole blood (50 mL) were collected from the jugular vein of a Bactrian camel. The inhibin α protein was diluted to a concentration of 1 mg/mL and then mixed with an equal volume of adjuvant to create an emulsified immunogen suspension. For the first immunization, Freund’s complete adjuvant was used, while Freund’s incomplete adjuvant was employed for subsequent immunizations. The emulsified immunogen was subcutaneously injected into camels. The interval between the first and second immunizations was set at 2 weeks, and the interval for subsequent immunizations was shortened to 1 week. On the third day following the third and sixth immunizations, whole blood samples were collected. The collected blood was then separated to obtain serum, and the antibody titers in the serum were measured to assess the immune response. On the third day after the last immunization, 100 mL of both non-anticoagulant and anticoagulant blood were collected for further analysis. The non-anticoagulant serum was collected each time through centrifugation. The antibody titer was determined using the inhibin α protein as the antigen. Then, each well of a 96-well enzyme plate was coated with 0.4 µg antigen and incubated overnight at 4 °C. The serum diluted through a 2-fold gradient was the first antibody, and the serum before immunization was used as the negative control. HRP-labeled mouse anti-camel serum IgG was used as a secondary antibody, and indirect ELISA was performed to determine the titer of the inhibin α antibody in Bactrian camels.

### 2.5. High-Flux Sequencing and Mass Spectrometry Analysis

The peripheral lymphocytes of whole blood (100 mL) from the Bactrian camels were extracted using a camel peripheral blood lymphocyte extraction kit before and after immunization. The RNA of the lymphocytes was extracted using the phenol-chloroform method and reverse transcribed into cDNA using the HiFiScript cDNA Synthesis Kit (Jiangsu CoWin Biotech Co., Ltd., Taizhou, China). The VHH cDNA of the Bactrian camel was amplified through nested PCR, and the primers are listed in Table 2 (GenBank accession number KU555411.1). The first round of PCR was performed using cDNA as a template and Call001 and Call002 as upstream and downstream primers, respectively (The partial cDNA sequence from Camelus bactrianus encoding the immunoglobulin gamma 1b heavy chain was replicated). Following PCR product electrophoresis, a 700 bp gel product was recovered (The 900 bp product corresponded to a conventional antibody heavy chain, whereas the 700 bp product represented a heavy chain from the HCAb). The second round of PCR was performed by using gel recovery products as templates and *VHH*-F and *VHH*-R as upstream and downstream primers, respectively (The cDNA sequence that encodes for the variable domain of the nanobody’s immunoglobulin heavy chain was duplicated). After the second round of PCR was successful, the 700 bp products of the first round of PCR were sent to biological companies for high-throughput sequencing. Usearch10.0.240 software clustered the high-throughput sequencing results into operational taxonomic units (OTU), and the VHH databases before and after immunization were obtained.

The inhibin α protein was used as an antigen. Then, each well of a 96-well enzyme plate was coated with 0.4 μg inhibin α protein. On the second day, after washing, the antigens in each well were blocked with 5% skim milk powder for 2 h at 37 °C. After the plate was washed with PBST, 100 μL of immunized serum was added to the enzyme plate. The plate was incubated at 37 °C for 2 h. After washing away unbound serum, the IgG that specifically binds to the inhibin α protein in the enzyme plate was scraped off with a plastic scraper and resuspended in PBS. Then, 5× SDS-PAGE loading buffer (containing a reducing agent) was added, and the samples were heated before polyacrylamide gel electrophoresis. The bands in the gel were excised for mass spectrometry. First, the SDS-PAGE gel after electrophoresis was subjected to the decolorization process, which included the steps of fixation, decolorization, and scanning, to obtain a clear image of the protein bands. Subsequently, the target protein bands were subjected to reduction and alkylation treatments, followed by enzymatic digestion with trypsin to extract the protein fragments and dry them. The redissolved peptide fragments were then separated through liquid chromatography (LC). Subsequently, mass spectrometry analysis was performed using a tandem mass spectrometry (MS/MS) system. The redissolved peptide fragments were then separated through LC (The mass spectrometry equipment used in this study includes the following: Mass spectrometer: Thermo Scientific Q-Exactive HF-X (Thermo Fisher Scientific, Waltham, MA, USA), Chromatography system: Easy-nLC1200, Thermo Scientific, Trap column: Reverse-phase, 100 µm × 20 mm (5 µm, C18). Analytical column: Thermo Scientific EASY column, Reverse-phase, 75 µm × 150 mm (3 µm, C18)). Subsequently, mass spectrometry analysis was performed using a tandem mass spectrometry (MS/MS) system. The selected peptide ion fragments from the primary mass spectrometric data were subjected to collision-induced dissociation or other fragmentation techniques to obtain fragment ions of the peptides. The sequence information of the peptides was acquired through secondary mass spectrometry analysis. Mass spectrometric data were successfully acquired for both pre-immunization and post-immunization serum samples, as well as for the inhibin α-specific antibody (The detailed experimental procedure is shown in File S1).

### 2.6. Screening and Gene Synthesis of Nanobodies

The VHH sequence alignment of the pre-immunization and post-immunization databases was performed by Mingke Biotechnology (Hangzhou, China) using a custom Perl program developed in-house. The unique OTU sequences identified in the post-immunization database were organized and compiled to form a database known as the *VHH* differential gene database, which contained new or altered *VHH* gene sequences that appeared after the immune response was generated. Using EMBOSS 6.6.0.0 software, the DNA sequences in the *VHH* differential gene database were translated into amino acid sequences, which resulted in a database containing VHH differential amino acid sequences. To facilitate subsequent search and analysis, each amino acid sequence must be associated with the name of its corresponding DNA sequence, ensuring that each amino acid sequence can be traced back to its original DNA sequence.

The mass spectrometric data was preprocessed, which included the steps of baseline correction, peak detection, charge state identification, and retention time alignment. Subsequently, the database search was conducted using MaxQuant 1.6.1.0 software (the database is the aforementioned VHH differential amino acid sequence database). Supplementary Table S1 presents the analysis parameters for the MaxQuant search engine. Following the mass spectrometric data search, the filtering criteria for the peptide, site, and protein identifications were set at PSM FDR ≤ 0.01 and protein FDR ≤ 0.01, respectively. Supplementary Table S2 presents the final statistics regarding the number of proteins and peptides identified in the samples.

Given that the complementarity-determining regions 1 (CDR1), CDR2, and CDR3 of nanobodies are approximately located at positions 24–36, 50–65, and 93–115 of the full nanobody sequence, respectively [27,28], further screening for inhibin α-specific antibodies was conducted by referring to their peptide libraries and post-immunization specific antibody protein libraries. Finally, the selected proteins were matched with genes in the *VHH* differential gene database to obtain the final inhibin α-specific nanobody gene sequences. Subsequently, the selected VHH sequences were aligned using Mega7 software, and a phylogenetic tree was constructed to further analyze their evolutionary relationships and structural characteristics.

After the suitable restriction site and E-tag gene sequence were added to the selected 7 VHH sequences, the sequences were sent to General Biology Co., Ltd. (Chuzhou, China) for gene synthesis. Then, the sequences were ligated to the pMD19-T vector and transformed into the *E. coli* BL21 and DH5α competent cells.

### 2.7. Construction of Nanobody Gene Vectors and Protein Expression

The *E. coli* sent from the company was cultured, and a plasmid extraction kit was used to extract plasmids from the *E. coli*. The recombinant plasmids containing the 7 nanobody genes were digested with suitable restriction endonucleases (Cloning Sites *Nde* I-*Not* I). The products obtained were double digested with enzymes for agarose gel electrophoresis, and the target gene was recovered using the gel recovery kit. The pET32a (+) plasmid was also double-digested with endonuclease, thereby recovering the target fragment. Then, T4 DNA ligase was used to ligate the target gene and the 32a vector. Following ligation, the recombinant plasmid was transformed into the *E. coli* BL21 (DE3) cells, and the positive transformants were screened on LB solid medium (containing ampicillin antibiotics). The selected positive transformants were identified through PCR, double enzyme digestion (Digested with *Apa* I-*Xho* I), and sequencing.

The positive strains were cultured at 37 °C and 170 r/min to the logarithmic growth phase and induced to express by IPTG. Samples of 1 mL bacterial culture media induced for 0, 2, 4, 6, and 8 h were collected, and polyacrylamide gel electrophoresis was performed. The remaining bacterial culture medium was centrifuged to obtain the pellet. The pellet was then resuspended in PBS, repeatedly freeze-thawed, and ultrasonically lysed. The crushed products were centrifuged for 20 min at 4 °C and 10,000 r/min. The supernatants and precipitates obtained were analyzed through polyacrylamide gel electrophoresis to determine the best induction time and expression form of recombinant proteins.

The pellet obtained through ultrasonic fragmentation was resuspended in inclusion body solution A (ibid.) and dissolved at 4 °C for 16 h. After the precipitate was dissolved, the mixture was centrifuged at 10,000 rpm for 20 min. The supernatant was collected, filtered through a 0.45-μm filter membrane, and added to the Ni+ agarose gel column. The nanobody proteins in the gel column were eluted with B solution (20 mM Tris–HCl, 500 mM imidazole, 0.5 M sodium chloride, and 8 M urea). Finally, urea concentrations of 6, 4, 2, and 1 M, followed by those of PBS, were used to sequentially dialyze the protein for 6–8 h. The protein was renatured, and polyacrylamide gel electrophoresis was performed.

### 2.8. Western Blot Identification of Nanobody Proteins

First, polyacrylamide gel electrophoresis of the purified nanobody protein was performed. Then, the gel region containing 15 kDa proteins was excised, and the protein was transferred to the PVDF membrane. The membrane was incubated with 5% skimmed milk powder at room temperature for 2 h. After the membrane was washed with TBST, it was incubated with the diluted mouse anti-His tag polyclonal antibody at room temperature for 2 h. The membrane was washed again and incubated with the diluted HRP-labeled goat anti-mouse polyclonal antibody at room temperature for 2 h. The membrane was washed and incubated in an ECL luminescent solution for 5 min to visualize the band.

### 2.9. Protein Simulation Docking and Affinity Identification

7 selected nanobodies were docked with inhibin α through protein–protein simulation. First, the AlphaFold 3 online software was used to predict the interactions between the 7 nanobodies and the inhibin α protein. Subsequently, PyMOL 3.1 software was employed to visualize the predicted results and analyze the interaction forces formed between the nanobodies and polar amino acid residues of inhibin α.

The inhibin α protein was used as an antigen, and then, each well of a 96-well enzyme plate was coated with 0.4 µg of the inhibin α protein. On the second day, 5% skim milk powder was used to block the plate for 2 h at 37 °C. Nanobodies diluted with a 2-fold gradient (250, 125, … and 0.122 μg/mL) were used as primary antibodies and incubated at 37 °C for 1 h. The nanobodies were incubated with the secondary antibody HRP-labeled mouse anti-E tag polyclonal antibody at 37 °C for 1 h. A 3,3′,5,5′-tetramethylbenzidine single-component chromogenic solution was used for 15 min at room temperature to develop color, followed by the addition of the stopping solution. The optical density (OD) value was measured at 450 nm.

### 2.10. Passive Immunity Test of Nanobodies in Female Mice

Twenty-eight healthy female Kunming mice (age: 8 weeks) with similar body weights were randomly assigned to the nanobody Nb-1737 immunization group, the nanobody Nb-1971 immunization group, the nanobody Nb-2004 immunization group, and the normal saline immunization group. All mice were immunized three times through intraperitoneal injection at an interval of 2 days between each immunization. The immunization dose administered each time was 500 μL, and the nanobody protein concentration was 300 μg/mL (the administration dosage was approximately 7.5 mg/kg). The control group was given an equivalent volume of normal saline. From the second day after immunization, the estrus cycle of the female mice was determined by vaginal smear examination every morning (9:00–10:00). Blood was collected via cardiac puncture and centrifuged to obtain serum, which was stored at −80 °C for subsequent hormone measurement. Serum levels of FSH, LH, inhibin A, and inhibin B were determined using commercial ELISA kits (Jiangsu Jingmei Biotechnology Co., Ltd., Yancheng, China) according to the manufacturers’ instructions. The mice in estrus were killed through cervical dislocation. Their ovaries, uterus, and pituitary tissues were collected after dissection. The number of ovarian follicles was counted (Ovarian tissues were fixed in Bouin’s solution, embedded in paraffin, sectioned, and stained with H&E for histological evaluation. Follicles in entire ovarian sections were counted by two blinded observers based on micrographs. The uterus was weighed by an investigator blinded to the group allocation; all visible fat and connective tissue were carefully dissected away before weighing, and the wet weight was normalized to body weight. RNA from the ovaries and pituitary tissues was extracted and reverse transcribed.

### 2.11. Passive Immunity Test of Nanobodies in Male Mice

Twenty-eight healthy male Kunming mice (8 weeks old) with comparable body weights were randomly divided into four groups: those immunized with nanobodies Nb-1737, Nb-1971, or Nb-2004, and a control group administered with saline. All mice received three intraperitoneal immunizations at 2-day intervals. Each injection consisted of a 500 μL dose containing nanobodies at 300 μg/mL, while the control group received an equal volume of normal saline. On the second day post-immunization, all male mice were euthanized by cervical dislocation at noon. Testes and pituitary tissues were then collected, and testicular wet weight was recorded. Subsequently, total RNA was extracted from the collected tissues and reverse-transcribed into cDNA.

### 2.12. Effect of Nanobody on Litter Size of Female Mice

Twenty-eight healthy female mice (8 weeks old, with similar body weights) were randomly assigned to four groups and immunized with nanobodies Nb-1737, Nb-1971, Nb-2004, or normal saline (control). All mice received three intraperitoneal injections at 2-day intervals. Each 500 μL injection contained nanobodies at a concentration of 300 μg/mL, while the control group received an equal volume of saline. Concurrently, 28 age- and weight-matched male mice were housed under the same conditions without treatment. On day 2 post-immunization, all mice were co-housed in pairs (one female and one male per cage). After 5 days, the male mice were removed, and the litter size of each female was subsequently recorded to evaluate the effects of nanobody immunization.

### 2.13. Detection of Gene Expression Levels

First, ovarian, testicular, and pituitary tissues were thoroughly washed with pre-cooled physiological saline to remove any potential impurities. Subsequently, a precise sample of 100 mg was taken from each tissue and ground into a fine powder in a low-temperature environment of liquid nitrogen (The processing and sampling of the detection samples were kept consistent, including the sampling site, time, sample size, growth conditions, and genetic background, to reduce the experimental result deviations caused by sample differences), followed by RNA being extracted from the tissues by using the Trizol method. Once extraction was complete, RNA was transcribed into cDNA through reverse transcription.

To ensure the accuracy of the quantitative polymerase chain reaction (qPCR) results, we first validated the specificity of the primers and the quality of the cDNA templates. Two reference genes were used: *GAPDH* and *β*-actin, and they yielded consistent results; for brevity, only *GAPDH* is reported in the main text. The specific procedure involved amplifying the cDNA extracted from the ovarian and testicular tissues by using *Inha*-qPCR-F and *Inha*-qPCR-R primers, as well as *GAPDH*-F and *GAPDH*-R primers. Concurrently, we amplified the cDNA extracted from the pituitary tissues by using *Fshb*-qPCR-F and *Fshb*-qPCR-R primers, as well as *GAPDH*-F and *GAPDH*-R primers. We then sequenced all PCR products for verification. Table 3 presents the details of the specific qPCR primer sequences, designed for the *Inha* gene (NM_001329843.1), the *Fshb* gene (NM_008045.3), and the *β*-actin gene (NM_007393.5) in addition to the *GAPDH* gene (GU214026.1).

The cDNA concentration from all tissues was uniformly diluted to 100 ng/μL to facilitate qPCR (Each sample was subjected to three replicates). In this experiment, we detected *Inha* gene transcription levels in the testes and ovaries, as well as *Fshb* gene expression in the pituitary. The *GAPDH* gene served as the reference gene. The qPCR reaction system totals 20 µL, with the specific composition as follows: cDNA: 2 µL, upstream and downstream primers: 1 µL each, qPCR Mix: 10 µL, dd H_2_O: 6 µL. The qPCR reaction conditions were set as follows: initial denaturation at 94 °C for 30 s, followed by 40 cycles of amplification, each involving denaturation at 94 °C for 5 s, annealing at 50 °C for 15 s, and extension at 72 °C for 10 s.

### 2.14. Statistical Analyses

The aforementioned data were statistically analyzed through one-way/two-way ANOVA using GraphPad Prism 9.0 software. Normality was checked using the Shapiro–Wilk test. A *p*-value < 0.05 was considered statistically significant.

## 3. Results

### 3.1. Preparation of Inhibin α Antigen and Analysis of Immune Response in Bactrian Camels

**Figure 1:**
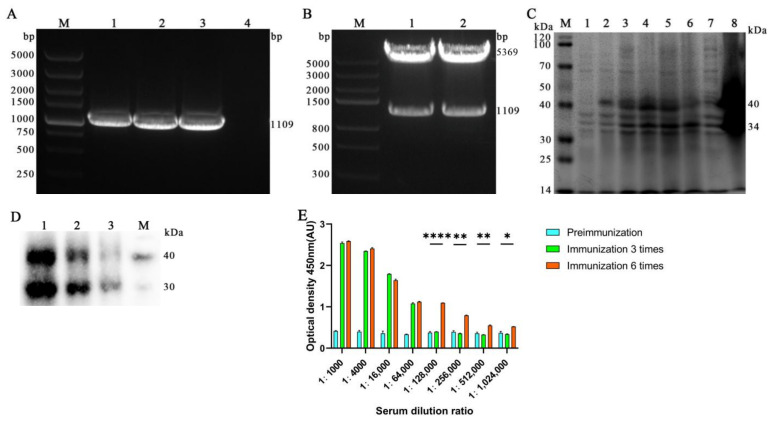
Preparation of Inhibin α Antigen and Analysis of Immune Response in Bactrian Camels. (**A**) Identification of the recombinant plasmid pET32a-*INHA* by PCR. M: DNA marker; Lanes 1–3: PCR products from the pET32a-*INHA* plasmid; Lane 4: Negative control. (**B**) Identification of the recombinant plasmid pET32a-*INHA* by double enzyme digestion. M: DNA marker; Lanes 1–2: Digestion products of the pET32a-*INHA* plasmid. (**C**) Analysis of the recombinant inhibin α protein expression by SDS-PAGE. M: Protein marker; Lanes 1–6: Protein expression induced for 0, 2, 4, 6, 8, and 10 h, respectively; Lanes 7–8: Supernatant and precipitate of the bacterial lysate after ultrasonication. (**D**) Western blot analysis of the purified inhibin α protein. Lanes 1–3: purified protein with loading amounts of 15, 10, and 5 μL, respectively; M: Protein marker. (**E**) Antibody titers in Bactrian camels after the third and sixth immunization. Data were analyzed by two-way ANOVA followed by Tukey’s post hoc test using GraphPad Prism. * *p* < 0.05, ** *p* < 0.01 and **** *p* < 0.0001.

After the pET32a-*INHA* vector-carrying strain was cultured, the bacterial liquid was subjected to PCR. According to the agarose gel electrophoresis results of PCR products, a single band was produced at the 1109 bp site (Figure 1A). As expected, the sequencing results were consistent with the GenBank sequence (NM_001308579.1). The plasmids extracted from the strain were double-digested with endonuclease, and the products were separated through agarose gel electrophoresis. The results revealed two bands of 5369 and 1109 bp (Figure 1B), consistent with the expected results. Thus, the prokaryotic expression vector of the *INHA* gene was correct.

SDS-PAGE analysis was performed on bacterial cultures induced for varying durations, as well as on the supernatant and pellet fractions obtained following ultrasonic cell disruption. The results revealed protein bands at approximately 34 kDa and 40 kDa corresponding to expression of the *INHA* gene (The theoretical molecular weight of the protein is 39 kDa) (Figure 1C), which differed from the theoretical molecular weight predicted from the gene sequence. The electrophoretic profile indicated that inhibin α protein expression peaked after 6 h of induction and that the protein was predominantly expressed in the form of inclusion bodies. Following purification using Ni^+^-agarose affinity chromatography, the protein sample still showed two major bands at 34 kDa and 40 kDa under denaturing electrophoretic conditions; Western blot analysis confirmed that both bands carried the His-tag (Figure 1D). To further verify their identity, the two bands were excised and subjected to LC-MS/MS analysis, which confirmed that both the 34 kDa and 40 kDa bands contain peptides uniquely matching the inhibin α protein sequence (Files S2 and S3). These results demonstrate that the recombinant protein stably exists in two distinct molecular weight forms during expression, which may be associated with common prokaryotic expression phenomena such as aberrant translation initiation or specific proteolytic cleavage.

Antibody titers in serum samples collected after the third and sixth immunizations were measured by indirect ELISA. Serum obtained after the third immunization showed no significant difference from pre-immunization serum at a dilution of 1:128,000. In contrast, serum following the sixth immunization remained markedly different from the pre-immunization control even at a dilution of 1:1,024,000. The antibody titer after the sixth immunization thus reached 1:1,024,000 (Figure 1E), supporting the progression to subsequent experimental stages.

### 3.2. Results of High-Throughput Sequencing and Mass Spectrometry Analysis

**Figure 2:**
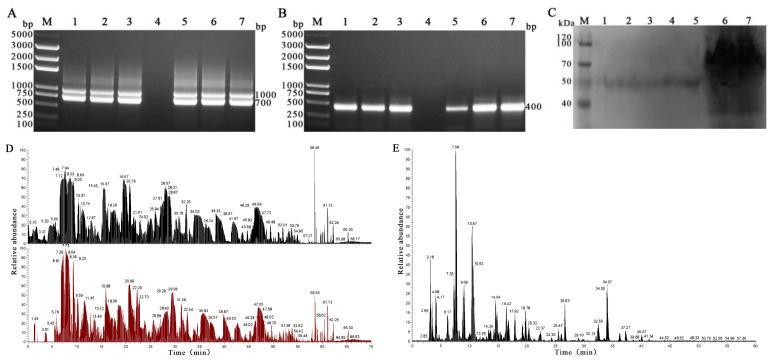
Results of high-throughput sequencing and mass spectrometry analysis. (**A**) Amplification of lymphocyte cDNA by first-round PCR. M: DNA marker; Lanes 1–3: Products from pre-immune samples; Lane 4: Negative control; Lanes 5–7: Products from post-immune samples. (**B**) Amplification of lymphocyte cDNA by second-round PCR (nested PCR). M: DNA marker; Lanes 1–3: Products from pre-immune samples; Lane 4: Negative control; Lanes 5–7: Products from post-immune samples. (**C**) Electrophoretic results of specific antibody screening products of inhibin α. M: protein marker; 1–5: Electrophoretic results of an inhibin α-specific antibody; 6–7: pre-immunization and post-immunization sera. (**D**) LC-MS chromatograms of serum proteins. Black line: pre-immune serum; Red line: post-immune serum. (**E**) LC-MS analysis of the purified inhibin α-specific antibody.

PCR was performed on the cDNA before and after immunization. After the first round of PCR electrophoresis, two bands of 1000 and 700 bp were observed. The 700 bp band was a heavy chain antibody sequence (Figure 2A). After the second round of PCR product electrophoresis, a 400 bp band (Figure 2B), which is the Bactrian camel nanobody (VHH) cDNA, was obtained. High-throughput sequencing confirmed the presence of 60,897 sequences in the pre-immunization VHH database. The total number of valid sequences was 57,841, with an average length of 332 bp (The average length was 368 bp after the primer sequences were added at both ends). After immunization, the VHH database contained 58,726 sequences. Of them, 53,994 sequences were valid, with an average length of 332 bp (The average length was 368 bp after the primer sequences were added at both ends).

Polyacrylamide gel electrophoresis revealed the presence of specific serum antibodies targeting inhibin α. A single band was produced at the 50 kDa site (Figure 2C), and the serum before and after immunization was difficult to distinguish because of the high protein concentration. The inhibin α-specific antibody was successfully screened from the serum. The gel block was excised and sent to Shanghai Baipu Biotechnology Co., Ltd. (Shanghai, China) for gel decolorization, protein enzymolysis, and LC-MS/MS analysis to obtain the LC-chromatograms (Figure 2D,E). The number of ion fragments obtained through ELISA screening were fewer, and the key ion fragments corresponded to the ion fragments before and after immunization. The results revealed that the inhibin α-specific antibody screened through ELISA was highly reliable.

### 3.3. Identification of Inhibin α-Specific Nanobodies

Pre-immunization and post-immunization VHH databases were constructed using sequences obtained through high-throughput sequencing. In total, 816 VHH cDNA sequences unique to the post-immunization VHH database were screened through database difference analysis, which is called the VHH differential gene database. In total, 4896 amino acid sequences were obtained by translating 816 VHH gene sequences into amino acids in the six reading frames, which is called the VHH differential amino acid database.

The mass spectrometric data of inhibin α-specific antibodies and sera (pre-immunization and post-immunization) were searched by referring to a mass spectrometry database. Subsequently, their peptide libraries were obtained. In total, 29 peptides (30 proteins) were retrieved from the mass spectrometric results of the inhibin α-specific antibodies (The detailed data are provided in Files S4 and S5). On analyzing the search results of the pre-immunization and post-immunization sera, 86 specific peptides (71 proteins) were screened from the post-immunization serum (The detailed data are provided in Files S6 and S7). Based on the position of the CDR of the nanobodies, ten peptides (10 proteins) were screened from 29 peptides of the inhibin α-specific antibody. Eight peptides (8 proteins) were selected from the aforementioned 86 specific peptides. Finally, 7 protein sequences with the highest score were selected based on the protein score in the mass spectrometry database. These proteins were located in the VHH differential gene database: OTU1712, OTU1971, OTU2000, OTU799, OTU2004, OTU1737, and OTU338. The first two of them were shared by the serum-specific protein library and the inhibin α-specific protein library after immunization.

**Figure 3:**
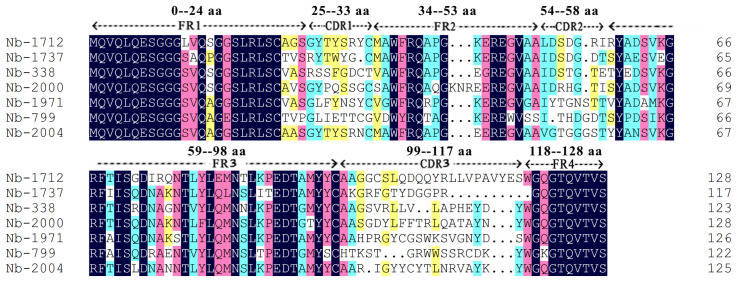
Comparison of amino acid sequences of 7 inhibin α-specific nanobodies. The CDR and FR annotation was performed according to the International Immunogenetics Information System (IMGT^®^).

The amino acid sequences of the 7 selected nanobodies (Nb-1712, Nb-1971, Nb-2000, Nb-799, Nb-2004, Nb-1737, and Nb-338) were compared (Figure 3). According to the alignment results, the overall similarity of the 7 amino acid sequences was 71.14%, with CDR1, CDR2, and CDR3 lengths of approximately 13, 13, and between 22 and 30, respectively. Among them, CDR3 exhibited the largest difference in length and sequence. Using these 7 sequences, a phylogenetic tree was constructed (Supplementary Figure S1), which revealed that the selected 7 nanobody amino acid sequences were rich in diversity.

### 3.4. Preparation of Nanobody Expression Vectors and Evaluation of Protein Production

**Figure 4:**
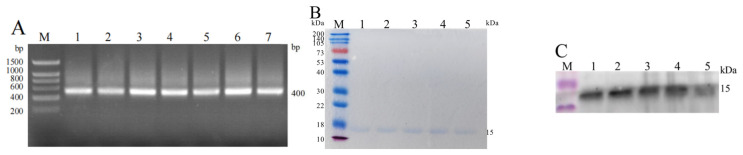
Preparation of nanobody expression vectors and evaluation of protein production. (**A**) Electrophoretic analysis of PCR products confirming nanobody gene insertion. M: DNA marker; Lanes 1–7: PCR amplification of the nanobody genes (Nb-338, Nb-799, Nb-1712, Nb-1737, Nb-1971, Nb-2000, and Nb-2004) from respective bacterial clones. (**B**) SDS-PAGE profile of the purified nanobody proteins. M: Protein marker; Lanes 1–5: Purified samples of nanobodies Nb-1712, Nb-1737, Nb-1971, Nb-2000, and Nb-2004. (**C**) Western blot analysis of the expressed nanobodies. M: Protein marker; Lanes 1–5: Detection of nanobodies Nb-1712, Nb-1737, Nb-1971, Nb-2000, and Nb-2004. The primary antibody used was mouse anti-His tag antibodies, and the secondary antibody used was HRP-labeled goat anti-mouse IgG.

To validate the constructed prokaryotic expression vectors for the 7 nanobody genes (pET32a-Nb), colony PCR was first performed on the corresponding bacterial strains using primers *VHH*-F and *VHH*-R (Table 2). A single band of approximately 400 bp was observed for all strains (Figure 4A). Subsequently, each recombinant plasmid was verified by double digestion with restriction enzymes. Electrophoretic analysis of the digested products showed two fragments of the expected sizes for every construct (Supplementary Figure S2), confirming the successful assembly of all 7 expression vectors.

Expression of the nanobody genes was induced with IPTG (Isopropyl β-D-1-thiogalactopyranoside). Bacterial cells were harvested at various time points after induction, lysed by sonication, and the resulting supernatant and pellet fractions were analyzed by SDS-PAGE. The results indicated that five of the 7 anobodies—Nb-1712, Nb-1737, Nb-1971, Nb-2000, and Nb-2004—were successfully expressed (Supplementary Figure S3). In contrast, expression of Nb-799 and Nb-338 was not detected, which may be due to a mismatch in codon usage preference with the host strain, potentially impairing translation efficiency. The five expressed nanobodies were purified using Ni^+^-agarose affinity chromatography. SDS-PAGE analysis of the purified proteins revealed a single band at approximately 15 kDa for each (Figure 4B), indicating that the target proteins were obtained at high purity and with the correct molecular weight.

Finally, Western blot analysis of the 5 purified nanobodies confirmed their identity, with each showing a clear band at around 15 kDa (Figure 4C). These results collectively verify the successful expression and effective purification of the nanobody proteins.

### 3.5. Molecular Docking and ELISA Analysis of Nanobodies Targeting the Inhibin α Protein

**Figure 5:**
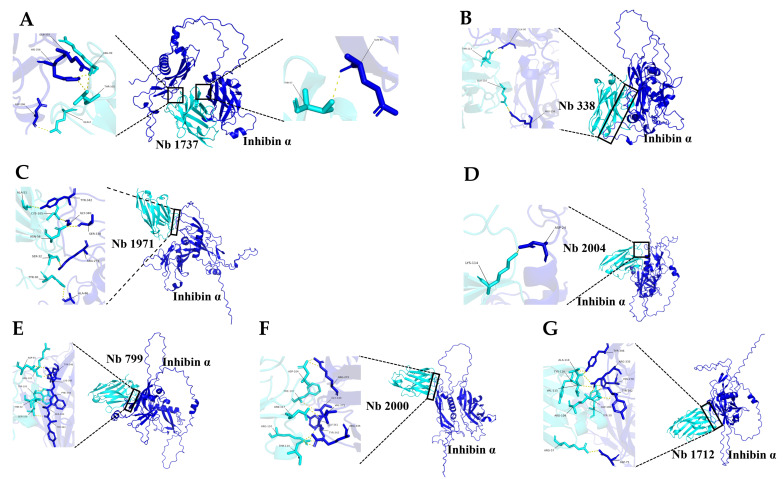
Molecular docking analysis of inhibin α with nanobodies. Predicted binding modes between inhibin α and nanobodies (**A**) Nb-1737, (**B**) Nb-338, (**C**) Nb-1971, (**D**) Nb-2004, (**E**) Nb-799, (**F**) Nb-2000, (**G**) Nb-1712.

Molecular docking of the seven nanobodies with inhibin α was performed using AlphaFold. The predicted binding modes are shown in Figure 5A–G, with the predicted interaction forces primarily concentrated in the CDR3 regions of the nanobodies (Table 4). The predominant interaction forces were predicted to be hydrogen bonds. However, the confidence of these docking models was evaluated using the ipTM (interface predicted Template Modeling) score, which ranges from 0 to 1. As shown in Table 4, the ipTM scores for all seven nanobody–inhibin α complexes ranged from 0.11 to 0.27, all well below the acceptable threshold. Therefore, these predicted binding modes are of low confidence and cannot be used to infer relative binding affinities or to rank the nanobodies. The models are presented solely as qualitative visual representations of potential interaction geometries. All conclusions regarding binding strength are based exclusively on the experimental indirect ELISA results described below.

**Figure 6:**
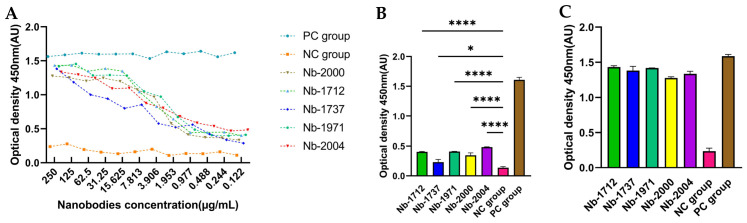
Analysis of nanobody binding affinity to inhibin α. (**A**) Representative results from ELISA showing the binding affinity of five nanobodies each to inhibin α. (**B**,**C**) Quantitative comparison of the affinity for five nanobodies at 250 μg/mL (**B**) and 0.122 μg/mL (**C**). PC stands for positive control, and NC stands for negative control. Data were analyzed by one-way ANOVA followed by Tukey’s post hoc test using GraphPad Prism. * *p* < 0.05 and **** *p* < 0.0001.

ELISA-based affinity analysis confirmed the binding capability of all five expressed nanobodies to the inhibin α subunit (Figure 6A). The binding curves of Nb-1712 and Nb-1971 closely followed a classical sigmoidal profile, indicative of specific antigen–antibody interaction kinetics. Notably, even at a low concentration of 0.122 μg/mL, nanobodies Nb-2004, Nb-1712, and Nb-1737 retained pronounced binding activity (Figure 6B,C). The assay exhibited high sensitivity and specificity, with optical density values >1.5 for positive controls and <0.3 for blanks. Both intra-assay and inter-assay coefficients of variation were low (1.86% and 2.71%, respectively), well below the commonly accepted thresholds of 10% and 15%, confirming excellent reproducibility and reliability of the affinity measurements.

### 3.6. Results of Passive Immunization in Mice

**Figure 7:**
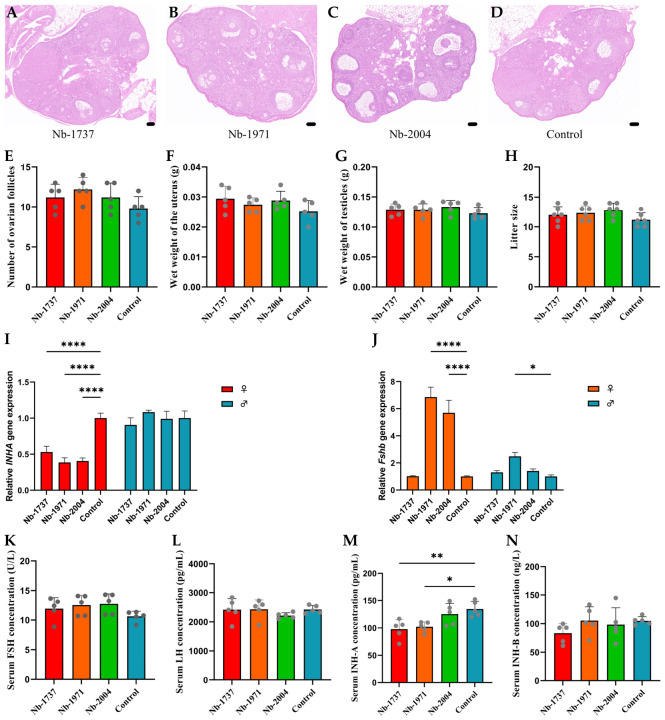
Evaluation of passive immunization in mice. (**A**–**D**) HE-stained sections of mouse ovary, Scale bars, 0.100 mm. (**E**) Quantification of ovarian follicles in female mice (*n* = 5 per group). (**F**) Measurement of uterine wet weight in female mice (*n* = 5 per group). (**G**) Measurement of testicular wet weight in male mice (*n* = 5 per group). (**H**) Analysis of litter size in female mice (*n* = 6 per group). (**I**) Relative expression levels of the *Inha* gene (*n* = 5 per group). (**J**) Relative expression levels of the *Fshb* gene (*n* = 5 per group). (**K**–**N**) Serum FSH, LH, INH-A, and INH-B levels by group (*n* = 5 per group). Data were analyzed by one-way/two-way ANOVA followed by Tukey’s post hoc test using GraphPad Prism. * *p* < 0.05, ** *p* < 0.01 and **** *p* < 0.0001.

Beginning on the second day after immunization, female mice in estrus were euthanized each morning, and their ovaries and uteri were collected. Ovarian follicles were quantified on hematoxylin and eosin (H&E)-stained histological sections, and uterine wet weight was recorded. The number of follicles was higher in all nanobody-treated groups compared with the saline control group (Figure 7A–E). All three nanobodies effectively promoted follicular development. Although uterine weight showed a slight increase in the nanobody groups relative to the control, the difference was not statistically significant (*p* > 0.05) (Figure 7F).

Following immunization, testes were collected from male mice and weighed. Testicular weight showed a slight increase in all nanobody-immunized groups relative to the saline control group, although the differences were not statistically significant (Figure 7G). All three nanobodies exhibited a tendency to promote testicular development, with Nb-2004 showing the most pronounced effect among the tested candidates. Furthermore, relative to the saline control group, female mice in all nanobody-immunized groups exhibited some increase in litter size, with an average increase of about 10%, and the Nb-2004 group showed the largest increase of 14.93% (Figure 7H). Among the nanobodies tested, passive immunization with Nb-2004 showed the strongest trend toward improving litter size. The lack of statistical significance may be attributed to the relatively short in vivo half-life of the monovalent nanobodies, which could limit the duration of their biological effects.

PCR amplification and sequencing confirmed the high specificity of the primers for all target and reference genes, as well as the reliable quality of the cDNA templates, thereby establishing a solid foundation for subsequent qPCR analysis (Supplementary Figure S4A–C). The RT-qPCR results revealed that the *Inha* gene expression level decreased in the female mice from the nanobody immunization groups compared with the control group (saline), and significant differences were observed between the Nb-1737 (*p* < 0.0001), Nb-2004 (*p* < 0.0001), Nb-1971 (*p* < 0.0001), and control groups (Figure 7I). The *Fshb* gene expression levels increased, but only Nb-1971 and Nb-2004 exhibited significant differences in its expression levels (*p* < 0.0001) (Figure 7J). After the male mice were immunized, no difference was observed in *Inha* gene expression level between the nanobody immunization groups and the control group (Figure 7I). The *Fshb* gene expression level increased, but only Nb-1971 exhibited significant differences (*p* < 0.01). No difference was observed in the other groups (*p* > 0.05) (Figure 7J).

Serum hormone analysis indicated elevated FSH levels in all nanobody-treated groups compared to the saline control, although the increases were not statistically significant (Figure 7K). LH levels remained comparable across all groups (Figure 7L). Notably, inhibin A levels were significantly suppressed in the Nb-1737 and Nb-1971 groups relative to the control (Figure 7M). For inhibin B, a decreasing trend was observed in the Nb-1737 group, but it did not reach statistical significance, and no differences were noted in the other treatment groups (Figure 7N).

## 4. Discussion

**Figure 8:**
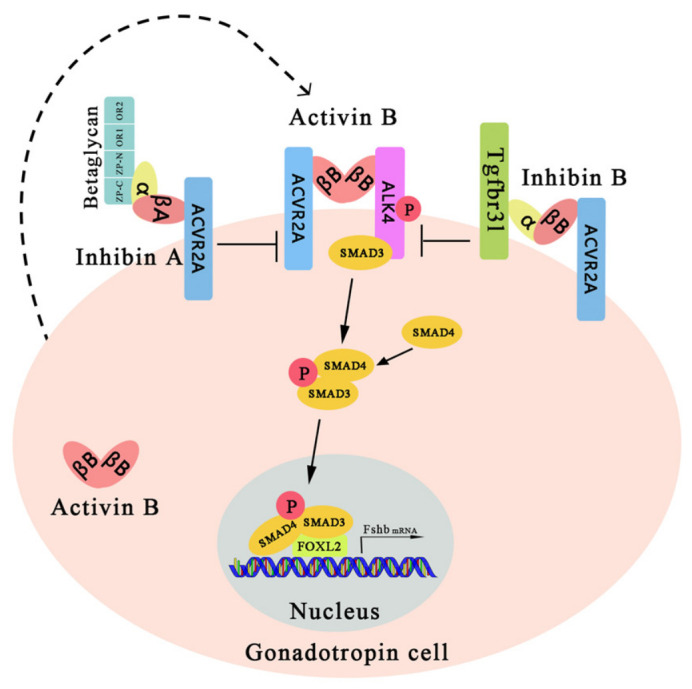
Schematic diagram of the regulatory mechanism of inhibin, activin, and FSH. Activin B, produced by gonadotropes, binds to activin type II receptors (ActRIIA/B) and recruits the type I receptor ALK4 to form a ternary complex, which then phosphorylates Smad2/3. Smad3 binds to Smad4, and the Smad3-Smad4 complex, together with FOXL2, translocates to the nucleus to promote *Fshb* gene transcription and FSH synthesis. Inhibin A (α-βA) binds to its specific co-receptor betaglycan (TGFBR3), while inhibin B (α-βB) binds to TGFBR3L. These complexes competitively bind to ActRIIA/B, thereby blocking activin signaling and suppressing *Fshb* transcription.

Activin promotes *Fshb* mRNA synthesis in gonadotropin cells by forming ternary complexes with activin type II and type I receptors [29,30]. Activin is the main driving force for FSH synthesis [31,32]. Inhibin prevents activin from exerting its biological function by competitively binding to the activin type II receptor after binding to the coreceptor (the coreceptor of inhibin A is TGFBR3 [33,34], and the coreceptor of inhibin B is TGFBR3L [35]). Thus, it indirectly inhibits FSH synthesis (Figure 8). When the nanobody has sufficient affinity for the α-subunit, it can reduce the levels of inhibin A (α subunit + βA subunit) and inhibin B (α subunit + βB subunit) by competitively binding to the α-subunit. Moreover, even if the nanobody and α-subunit successfully bind to the β-subunit, they can block the binding of inhibin to its coreceptor, thereby preventing inhibin from exerting its biological function. We used protein simulation docking and indirect ELISA to identify the affinity of nanobodies to the inhibin α protein. Several nanobodies exhibited sufficient affinity toward inhibin α-subunits. Therefore, we predicted that these nanobodies can effectively prevent inhibin from exerting its biological function. We performed molecular docking using AlphaFold to visualize potential interaction modes. However, due to very low ipTM confidence scores (all <0.3; see Table 4), these docking models cannot reliably predict binding affinity or guide the selection of nanobodies. Therefore, the identification of high-affinity nanobodies was based exclusively on indirect ELISA. Based on the results of the indirect ELISA analysis, three nanobodies (Nb-1737, Nb-1971, and Nb-2004) with the best affinity were selected from among the 7 nanobodies for a follow-up passive immunity test to further identify the biological function of these nanobodies. The results show that these three nanobodies downregulated *Inha* and upregulated *Fshb* gene expression in mice, stimulated FSH secretion, promoted follicular development, and increased litter size, demonstrating their potential to regulate reproductive function.

The inhibin α protein is used as an antigen to immunize Bactrian camels, and its correct folding and high purity are critical for successful immunization [36,37]. In this experiment, the inhibin α protein was expressed as inclusion bodies, which may contain excess salt, endotoxins, and other harmful substances that are detrimental to animal health and immune function. Therefore, the protein was initially purified, treated with decreasing concentrations of urea (6, 4, 2, and 1 mol/L) for 6 h to break disulfide bonds, fully extend the peptide chain, and gradually renature the protein to restore its natural conformation [38], followed by dialysis against PBS (0.1 mol/L) for another 6 h to reduce impurities. During this process, we observed two protein bands (~34 kDa and ~40 kDa) on SDS-PAGE. Both bands were recognized by an anti-His antibody, confirming the presence of the C-terminal His-tag. To definitively identify these species, we performed LC-MS/MS analysis, which confirmed that both bands correspond to the inhibin α protein. The 34 kDa band is likely a degradation product or the result of aberrant translation initiation—a common phenomenon in prokaryotic expression of eukaryotic proteins. However, we must also acknowledge that the pET32a vector drives expression of the inhibin α protein in the bacterial cytosol, which is a reducing environment that does not support disulfide bond formation. The gradual urea treatment and dialysis performed here allow protein renaturation in terms of secondary and tertiary structure but do not promote the reformation of disulfide bridges. Therefore, we cannot claim to have fully restored the natural conformation of the inhibin α protein, particularly if disulfide bridges are critical for its native structure.

The nanobody of the Bactrian camel is composed of four frame regions and three CDRs arranged alternately. Genes in the frame region are highly conservative. Therefore, based on the frame regions at both ends of the nanobody gene, primers were designed for high-throughput sequencing. This technique can obtain all the nanobody genes in Bactrian camel lymphocytes and ensure data integrity. Other techniques, like phage display for nanobody selection, encompass procedures such as transformation and phage encapsulation. These steps can inadvertently constrain the diversity and complexity of the library, potentially leading to outcomes that may not completely meet expectations. For example, Kravchenko et al. [39] explicitly reported that the cloning step in phage display is a bottleneck limiting library diversity, leading to the loss of poorly represented variants before the selection procedure even begins. Heyduk [40] and others also experienced the problem of the selected antibodies being too single when the ribosome display technology was used for screening serum antibodies. In this study, through an integrated analysis of mass spectrometry data derived from the inhibin α-specific antibody, pre-immunization, and post-immunization sera, this approach effectively reduced the candidate nanobody gene pool while markedly improving screening accuracy. This provides a robust foundation for the efficient acquisition of inhibin α-specific nanobody genes.

Intraperitoneal immunization of mice with a specific inhibin α nanobody significantly downregulated ovarian *Inha* expression and upregulated pituitary *Fshb* expression. This subsequently stimulated FSH secretion, leading to enhanced follicular development and increased litter size. Because the FR2 region of the nanobody was rich in hydrophilic amino acids, the excellent water solubility of these amino acids facilitates intraperitoneal injection and ensures efficient drug delivery [41]. Intraperitoneal injection is simple and rapid and can allow administration of larger volumes of the drug, which is a major advantage [42,43]. The peritoneal cavity is lined by a vast peritoneum, which has an area comparable to that of the skin, thereby offering a huge absorption surface for the drug [41,44]. Peritoneal mesothelial cells are key in maintaining the stability of the peritoneal environment and the transmembrane transport of fluids and solutes. Below the peritoneum lies a dense and efficient network of blood and lymphatic vessels in the interstitial layer, which further promotes rapid drug absorption [45]. Therefore, as a drug administration method, intraperitoneal injection is convenient and effectively improves drug bioavailability. The relatively modest enhancement of reproductive parameters observed in this study may be largely due to the short in vivo half-life characteristic of monovalent nanobodies. This brief half-life likely compromised the duration of effective drug exposure and the maintenance of therapeutic plasma levels, thereby limiting the sustainability and overall potency of the effects. Similar pharmacokinetic constraints have been noted for nanobodies in prior studies [46,47].

The mass spectrometry data of some peptides were limited, characterized by weak ion sequences, a low signal-to-noise ratio (SNR), weak fragmentation, and low ion counts, which may affect the reliability of peptide assignment. Although these limitations did not shake the overall research conclusions, related peptides should be handled with caution in the final analysis. To improve data quality, we plan to use a higher-resolution mass spectrometer in the future, implement stricter search criteria, and manually verify assignments. According to the guidelines of Smith et al. [48], the SNR should be at least 10 to ensure the reliability of the ion assignment. However, the data of some peptides in the current study did not meet this standard, highlighting the necessity of adopting stricter criteria. Although the mass spectrometry limitations of some peptides did not affect the overall conclusion of the study, it emphasized the importance of using high-resolution mass spectrometers and strict data analysis standards to ensure the reliability of peptide assignments.

In gene quantification experiments, we observed differences in the expression of the *Inha* gene and *Fshb* gene between male and female mice, especially in the expression of the *Inha* gene. Specifically, nanobody immunization did not show differences in male mice, but significant differences were observed in female mice. These differences may be related to gender and tissue specificity. In female mice, the *Inha* gene is mainly expressed by ovarian granulosa cells, while in male mice, it is mainly expressed by testicular epithelial cells. In addition, the expression levels at different growth stages also vary. With some exceptions, adult male mammals usually only produce inhibin B [49]. Inhibin B is the main hormone form in the follicular phase of the female menstrual cycle and in the estrous/inter-estrous period of female rodents [50]. Both inhibin A and inhibin B are heterodimers composed of the inhibin α subunit and the inhibin βA and βB subunits, respectively. Therefore, there may be selective pressure to promote the expression of the *Inha* gene in specific genders and tissues to meet different physiological needs.

This study identified 7 nanobody genes specifically targeting the inhibin α subunit using high-throughput sequencing and mass spectrometry. Their high affinity was confirmed by indirect ELISA. Passive immunization in mice revealed that three of these nanobodies downregulated *Inha*, upregulated *Fshb*, and stimulated FSH secretion, thereby promoting follicular development. Although the increase in litter size was not statistically significant, these findings demonstrate the potential of these nanobodies to regulate reproductive function. This study establishes a foundation for developing nanobody-based strategies to improve animal fertility.

## 5. Conclusions

In this study, we successfully identified seven inhibin α-subunit-specific nanobodies from a Bactrian camel library using high-throughput sequencing combined with mass spectrometry. Five of these nanobodies were efficiently expressed in *E. coli* and showed high binding affinity to the inhibin α protein. Passive immunization experiments in mice demonstrated that three nanobodies—Nb-1737, Nb-1971, and Nb-2004—significantly downregulated ovarian *Inha* expression, upregulated pituitary *Fshb* expression, and stimulated FSH secretion, thereby promoting follicular development. Although a numerical increase in litter size (approximately 10%) was observed, the difference was not statistically significant, likely due to the short in vivo half-life of monovalent nanobodies. These findings provide a promising, low-cost alternative to conventional FSH-based ovarian stimulation and establish a foundation for developing nanobody-based passive immunization strategies to improve animal fertility. Future work should focus on extending nanobody half-life and evaluating their efficacy in larger animal models.

## Figures and Tables

**Table 1:** PCR primers for *INHA* gene.

Primers	Primer Sequences (5′→3′)	Annealing Temperature	Product Length	NM-Number
*INHA*- *Nde* I-F	GGAATTCCATATGATGTGGCTTCAGCTGCTCCTCTTC	53 °C	1106 bp	NM_001308579.1
*INHA*- *Not* I-R	ATAAGAATGCGGCCGCGATGCAAGCACAGTGCTGGGTG			

The 5′ restriction sites and protective bases are underlined.

**Table 2:** Nest PCR primers for *VHH* genes.

Primers	Primer Sequences (5′→3′)	Annealing Temperature	Product Length
Call001	GTCCTGGCTGCTCTTCTACAAAG	56.5 °C	700 bp
Call002	GGTACGTGCTGTTGAACTGTTCC		
*VHH*-F	CAGGTGCAGCTGCAGGAGTCT	58 °C	400 bp
*VHH*-R	TGAGGAGACGGTGACCTGGGT		

**Table 3:** qPCR primers.

Primers	Primer Sequences (5′→3′)	Annealing Temperature	Product Length	GenBank Accession
*Inha*-qPCR-F	CTTTCCCTCTGCTGACCCA	50 °C	184 bp	NM_001329843.1
*Inha*-qPCR-R	AAAGCCGCAGGAGACCAA			
*Fshb*-qPCR-F	CATCTTATTCTGGTGCTGG	50 °C	226 bp	NM_008045.3
*Fshb*-qPCR-R	AGGCAATCTTACGGTCTC			
*GAPDH*-F	CCTTCCGTGTTCCTACCC	50 °C	150 bp	NM_007393.5
*GAPDH*-R	CAACCTGGTCCTCAGTGTAG			
*β*-actin-F	AATCGTGCGTGACATCAA	50 °C	170 bp	GU214026.1
*β*-actin-R	AGAAGGAAGGCTGGAAAA			

**Table 4:** Predicted interaction sites and AlphaFold confidence scores (ipTM) for nanobody–inhibin α complexes.

Nanobody	CDR1	CDR2	CDR3	ipTM
Nb 338			GLU-110 (ARG-258); TYR-113 (GLN-80)	0.11
Nb 799	THR-32 (SER-343)	ASP-55 (LYS-345)	ARG-103 (TYR-346); TRP-105 (PHE-344); SER-106 (TYR-342)	0.11
Nb 1712	TYR-30 (HIS-276)		GLY-100, ALA-114, VAL-115 (ALG-335); ALA-114, TYR-116 (TYR-346); ALG-109 (TYR-342)	0.14
Nb 1737		THR-57 (GLN-80)	ARG-99 (HIS-356); THR-102 (GLN-355, HIS-356)	0.14
Nb 1971	TYR-30 (ALA-86); SER-32 (ALG-272)	ALA-51 (TYR-342); ASN-56 (SER-338)	CYS-105 (GLY-340)	0.2
Nb 2000			ASP-104 (ARG-272); PHE-107 (GLY-341); ARG-110 (GLY-341, TYR-342); THR-114 (ALG-335)	0.27
Nb 2004			LYS-114 (ASP-24)	0.12

Annotation: The table shows the predicted amino acid residues interacting between inhibin α and nanobody CDR regions (nanobody residues outside parentheses; inhibin α residues inside). ipTM scores range from 0 to 1 (scores > 0.8 indicate high confidence). All scores in this study were <0.3, indicating low confidence. Therefore, these predicted residues should be interpreted with caution and do not reflect binding affinity.

## Data Availability

The data generated and analyzed in this study will be stored in the GenBank database (Accession: PRJNA1066481 ID: 1066481). The data are anonymously accessible, and no special permissions are required.

## Supplementary Materials

The following supporting information can be downloaded at: https://www.mdpi.com/article/10.3390/ani16131961/s1, File S1: Detailed experimental procedure for mass spectrometry analysis; File S2: Summary of LC-MS/MS peptide identification results for the inhibin α protein; File S3: Summary of LC-MS/MS protein identification results for the inhibin α protein; File S4: Summary of LC-MS/MS peptide identification results for inhibin α-specific antibodies in pre-immune serum; File S5: Summary of LC-MS/MS peptide identification results for inhibin α-specific antibodies in post-immune serum; File S6: Summary of LC-MS/MS protein identification results for inhibin α-specific antibodies in pre-immune serum; File S7: Summary of LC-MS/MS protein identification results for inhibin α-specific antibodies in post-immune serum; Table S1: MaxQuant Analysis Parameter Settings; Table S2: Statistics of Protein Identification Outcomes; Figure S1: Evolutionary tree of amino acid sequences of 7 inhibin α-specific nanobodies; Figure S2: Electrophoretic results of double digestion products of 7 nanobody gene prokaryotic expression vectors. 1–2: Pre- and post-digestion products of the pET32a-Nb338 carrier (digestive site: *Apa* I-*Xho* I), 3–4: Pre- and post-digestion products of the pET32a-Nb1712 carrier (digestive site: *Apa* I-*Xho* I), 5–6: Pre- and post-digestion products of the pET32a-Nb799 carrier (digestive site: *Apa* I-*Xho* I), 7–8: Pre- and post-digestion products of the pET32a-Nb1737 carrier (digestive site: *Apa* I-*Xho* I), 9–10: Pre- and post-digestion products of the pET32a-Nb1971 carrier (digestive site: *Apa* I-*Xho* I), 11–12: Pre- and post-digestion products of the pET32a-Nb2000 carrier (digestive site: *Apa* I-*Xho* I), 13–14: Pre- and post-digestion products of the pET32a-Nb2004 carrier (digestive site: *Apa* I-*Xho* I), M: DNA marker; Figure S3: Results of induced expression electrophoresis of nanobodies. M: protein marker, 1–5: nanobody proteins were induced for 0, 2, 4, 6, and 8 h, respectively, 6–7: supernatant and precipitation of bacterial liquid after ultrasonic crushing; Figure S4: PCR product electrophoresis results of *INHA*, *Fshb*, and *GAPDH* genes. A. Result of *INHA* gene PCR product electrophoresis. M: DNA marker, 1–2: *INHA* gene PCR electrophoresis in ovarian tissue, 3–4: *INHA* gene PCR electrophoresis in testicular tissue. B. Result of *Fshb* gene PCR product electrophoresis. M: DNA marker, 1–2: *Fshb* gene PCR electrophoresis in male mouse pituitary, 3–4: *Fshb* gene PCR electrophoresis in female mouse pituitary. C. Result of *GAPDH* gene PCR product electrophoresis. M: DNA marker, 1–2: *GAPDH* gene PCR electrophoresis in ovarian tissue, 3–4: *GAPDH* gene PCR electrophoresis in testicular tissue. 5–6: *GAPDH* gene PCR electrophoresis in male mouse pituitary, 7–8: *GAPDH* gene PCR electrophoresis in female mouse pituitary.
