# Supplementary material for: Screening and Preliminary Identification of Inhibin α Subunit-Specific Nanobodies Through High-Throughput Sequencing Combined with Mass Spectrometry
Source: Animals (Basel). 2026 Jun 25;16(13):1961. doi: 10.3390/ani16131961 (PMC13360298; doi:10.3390/ani16131961)
Supplement: Supplementary file 1 [file animals-16-01961-s001.zip › File S1.pdf]

Experimental Reagents and Instruments:
(1) Ammonium bicarbonate (Sigma, A6141)
(2) Acetonitrile (Millipore, 1.00030.4008)
(3) Trypsin (Promega, V5113)
(4) Centrifuge (Eppendorf, 5430R)
(5) Pure water system (Thermo Scientific, GenPure)
(6) Mass spectrometer (Thermo Scientific, Q-Exactive Plus)
(7) Chromatography system (Easy-nLC1200, Thermo Scientific)
(8) Chromatography column (Trap column (Reverse-phase), 100μm*20mm (5um, C18))
(9) Chromatography analysis column (Thermo Scientific EASY column (Reverse-phase), 75μm*150mm (3um, C18))

Protein Digestion
Decolorization and Washing:
Add 800 μL of 0.1M NH_4HCO_3/30% ACN to the stained gel samples and mix with the gel pieces. Wash until the blue color of the protein disappears, then remove the supernatant.
Immediately add 800 μL of H_2O to terminate the reaction for 10 minutes, then remove the supernatant.
Reduction of Proteins:
Add 40 μL of 100 mM DTT and 360 μL of 100 mM NH_4HCO_3 to each sample. Incubate at 56°C for 30 minutes to reduce the proteins.
Remove the supernatant, add 100 μL of 100% ACN, and let it sit for 5 minutes before removing the liquid.
Alkylation of Proteins:
Add 280 μL of 100 mM NH_4HCO_3 and 120 μL of 200 mM IAA (freshly prepared, stored in the dark) to each sample. Incubate in the dark at room temperature for 20 minutes.
Remove the supernatant, add 100 μL of 100 mM NH_4HCO_3, and let it sit at room temperature for 15 minutes. Remove the supernatant again.
Add 100 μL of 100% ACN, let it sit for 5 minutes, then remove the liquid and lyophilize the sample.
Digestion with Trypsin:
Add 600 μL of 20 ng/μL Trypsin to each sample and place it in a 4°C refrigerator for about 30 minutes to allow the gel pieces to swell.
Add approximately 100 μL of 50 mM NH_4HCO_3 buffer, then incubate overnight at 37°C for digestion.
Aspirate the digestion solution and transfer it to a new centrifuge tube. Add 100 μL of 60% ACN/0.1% TFA (trifluoroacetic acid) to the gel pieces, sonicate for 15 minutes, aspirate the solution, and combine it with the previous solution. Repeat the extraction three times, combine the solutions, and lyophilize.
Desalting and Concentration:
Desalt the peptides using a C18 StageTip column and concentrate by drying.
Dissolve the peptides in a 0.1% formic acid aqueous solution for subsequent LC-MS/MS analysis.

LC-MS/MS Analysis

Sample Preparation:

Take an appropriate amount of peptides from each sample and use the Easy nLC 1200 chromatography system (Thermo Scientific) for chromatographic separation.

Buffer Solutions:

Buffer A: 0.1% formic acid aqueous solution.

Buffer B: 0.1% formic acid, acetonitrile, and water mixture (with 80% acetonitrile).

Chromatographic Conditions:

The chromatography column is balanced with 100% Buffer A.

The sample is injected into the Trap Column (100μm20mm, 5μm, C18, Dr. Maisch GmbH) and then separated through the analytical column (75μm150mm, 3μm, C18, Dr. Maisch GmbH) with a flow rate of 300 nl/min.

Gradient Elution:

0 minutes to 2 minutes: Linear gradient of Buffer B from 3% to 5%.

2 minutes to 42 minutes: Linear gradient of Buffer B from 5% to 25%.

42 minutes to 52 minutes: Linear gradient of Buffer B from 25% to 45%.

52 minutes to 55 minutes: Linear gradient of Buffer B from 45% to 90%.

55 minutes to 70 minutes: Buffer B maintained at 90%.

Mass Spectrometry Analysis:

After peptide separation, use the Q-Exactive Plus mass spectrometer (Thermo Scientific) for DDA (data-dependent acquisition) mass spectrometry analysis.

Analysis duration: 70 minutes.

Detection mode: Positive ion mode.

Parent ion scan range: 300-1800 m/z.

First-order mass spectrometry resolution: 70,000 @ m/z 200.

AGC target: 3e6.

First-order Maximum IT: 30 ms.

Second-Order Mass Spectrometry Analysis:

After each full scan, trigger the acquisition of MS2 scans for the 20 most intense parent ions.

Second-order mass spectrometry resolution: 17,500 @ m/z 200.

AGC target: 2e5.

Second-order Maximum IT: 60 ms.

MS2 Activation Type: HCD.

Isolation window: 1.6 m/z.

Normalized collision energy: 30.

Database Search

The mass spectrometry database search software used in this project is MaxQuant 1.6.17.0; the protein database used is from UniProt. The analysis parameters set for the MaxQuant search software are shown in Table S1.

Table S1 MaxQuant Analysis Parameter Settings

Item	Value
Enzyme	Trypsin
Max Missed Cleavages	2

Precursor Tolerance（Main search）	4.5 ppm
Precursor Tolerance（First search）	20 ppm
MS/MS Tolerance	20 ppm
Fixed modifications	Carbamidomethyl (C)
Variable modifications	Oxidation (M),Acetyl (Protein N-term)
Database	uniprot-Camelus [9836]-52909-20220128.fasta
PSM FDR	0.01
Protein FDR	0.01
Site FDR	0.01
