# Supplementary material for: Screening and Preliminary Identification of Inhibin α Subunit-Specific Nanobodies Through High-Throughput Sequencing Combined with Mass Spectrometry
Source: Animals (Basel). 2026 Jun 25;16(13):1961. doi: 10.3390/ani16131961 (PMC13360298; doi:10.3390/ani16131961)

OTU1712_1 3_65205

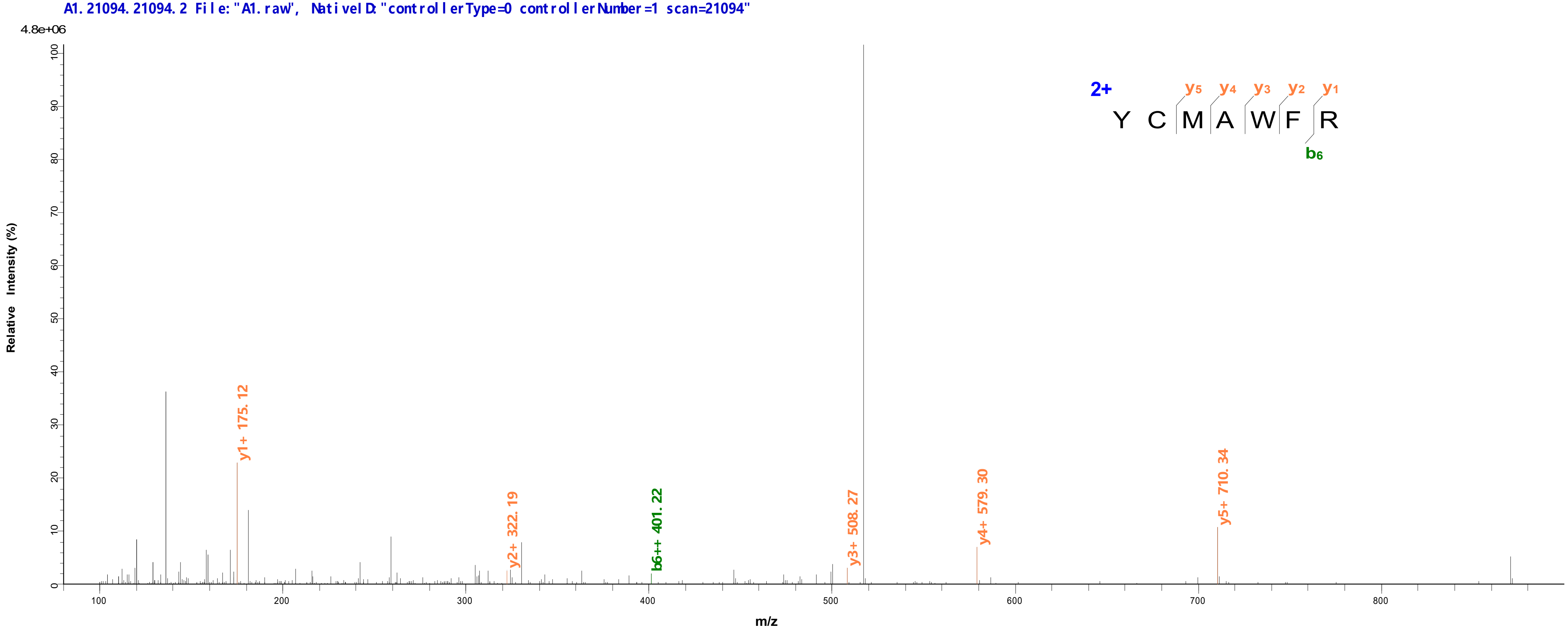

OTU1090_1 3_63152

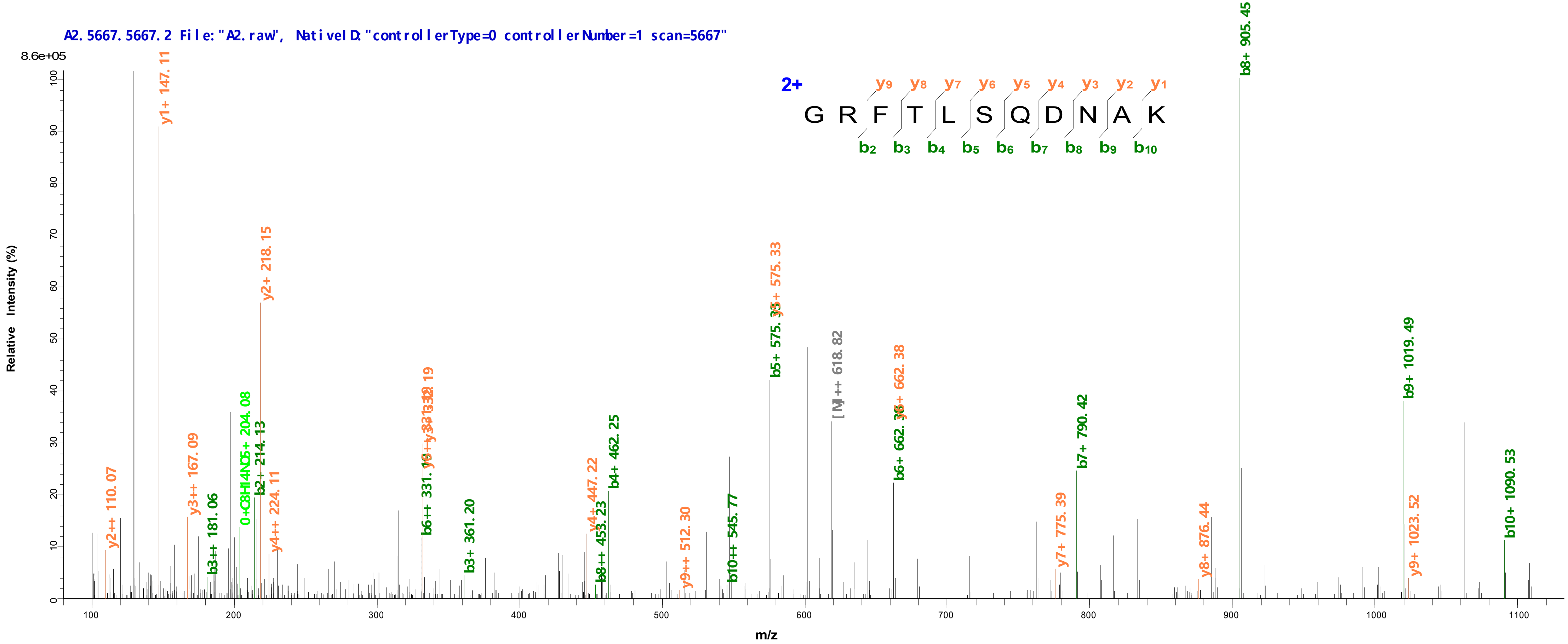

OTU267_1 3_97272

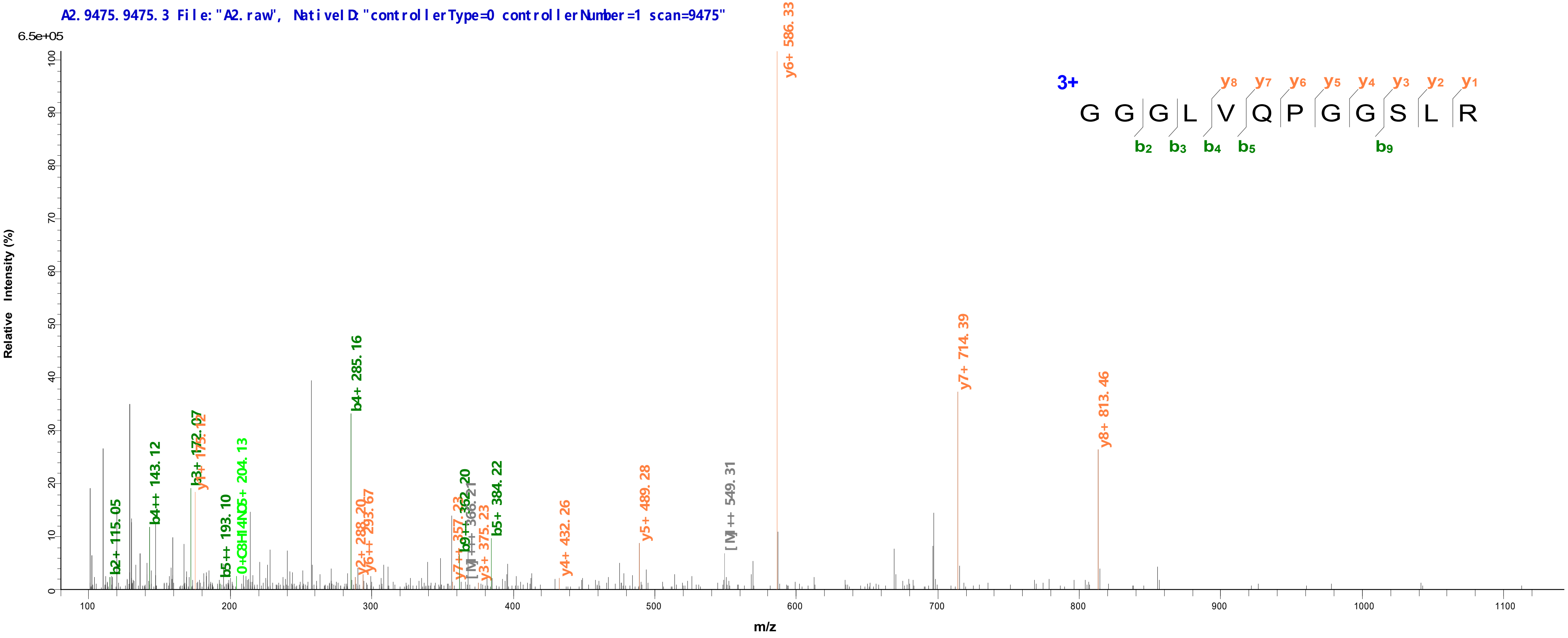

OTU1495_1 3_103647

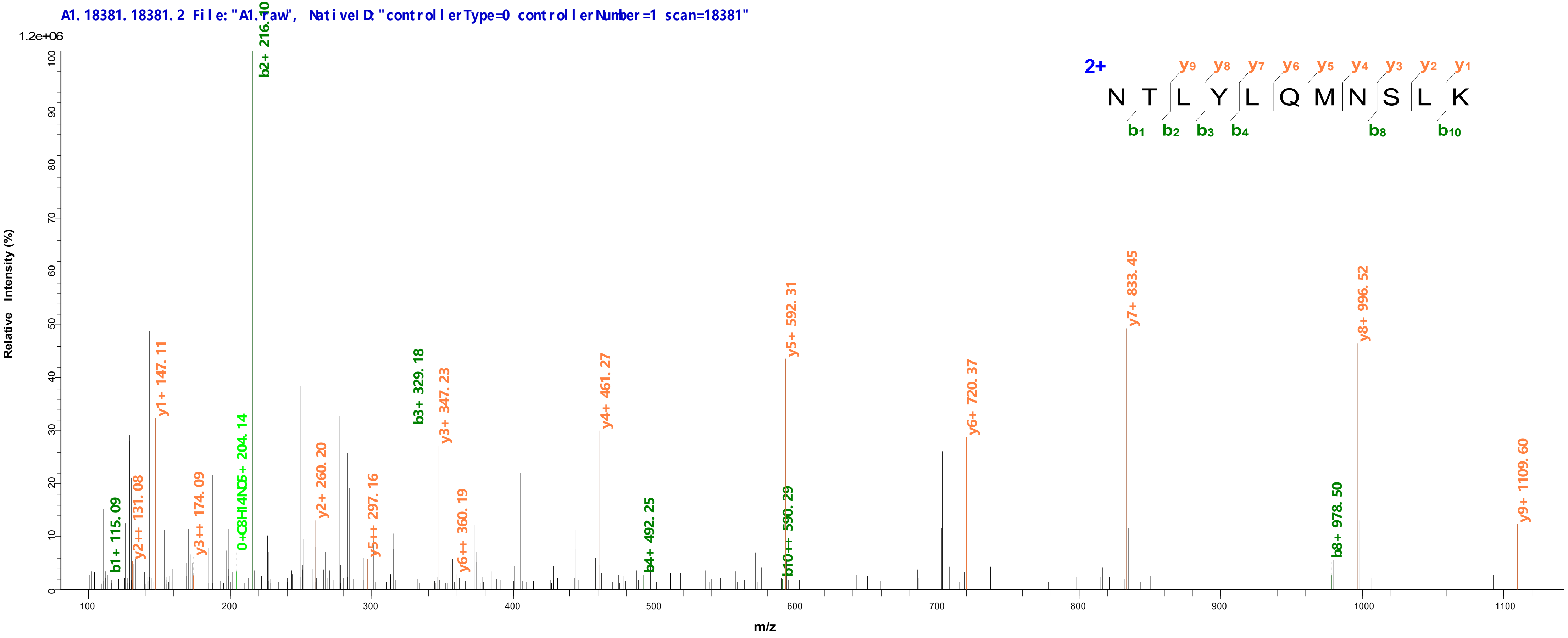

OTU1971_13_100830

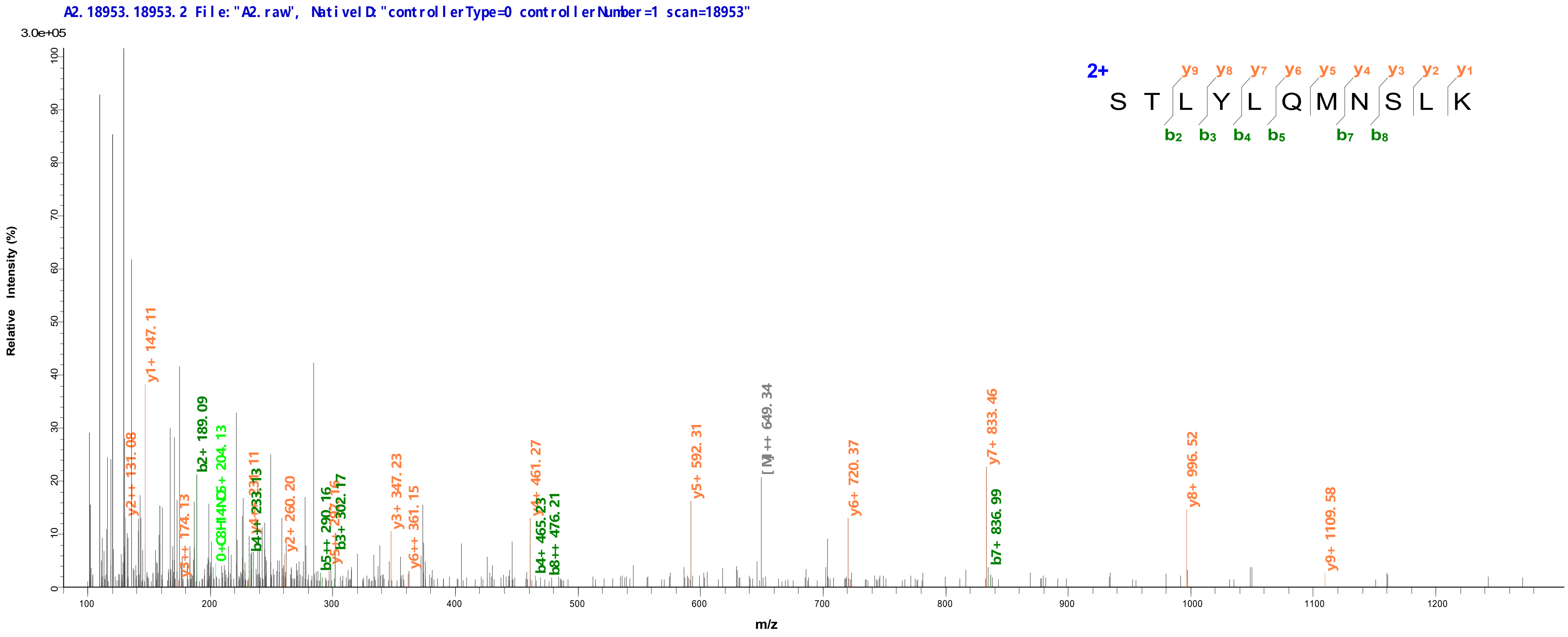

OTU573_13_106697

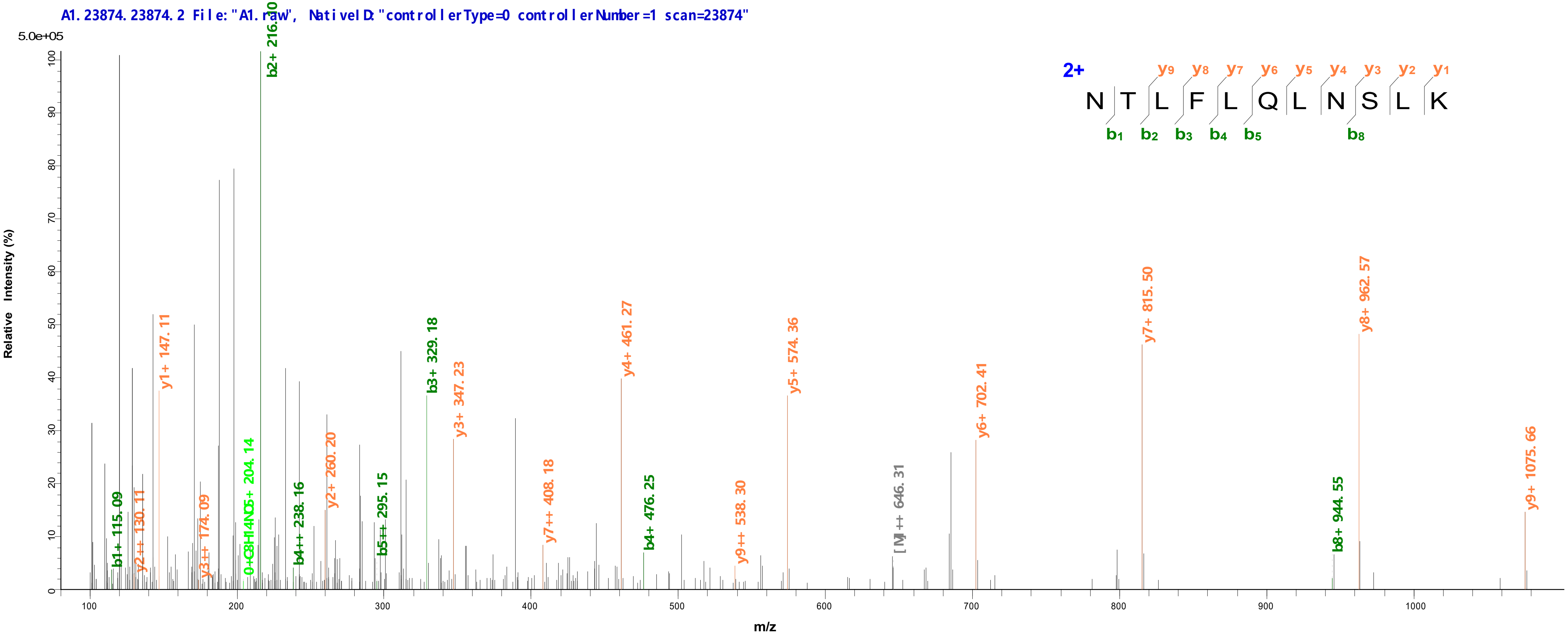

Supplement: Supplementary file 1 [file animals-16-01961-s001.zip › gas phase fragmentation spectra of peptides of specific antibodies after immunization.pdf]
