# Supplementary material for: Screening and Preliminary Identification of Inhibin α Subunit-Specific Nanobodies Through High-Throughput Sequencing Combined with Mass Spectrometry
Source: Animals (Basel). 2026 Jun 25;16(13):1961. doi: 10.3390/ani16131961 (PMC13360298; doi:10.3390/ani16131961)
Supplement: Supplementary file 1 [file animals-16-01961-s001.zip › Supplemental Figure.pdf]

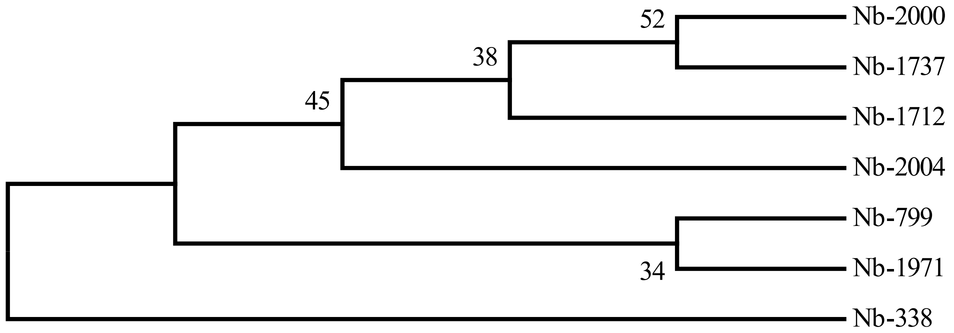

Supplemental Figure S1. Evolutionary tree of amino acid sequences of 7 inhibin α-specific nanobodies.

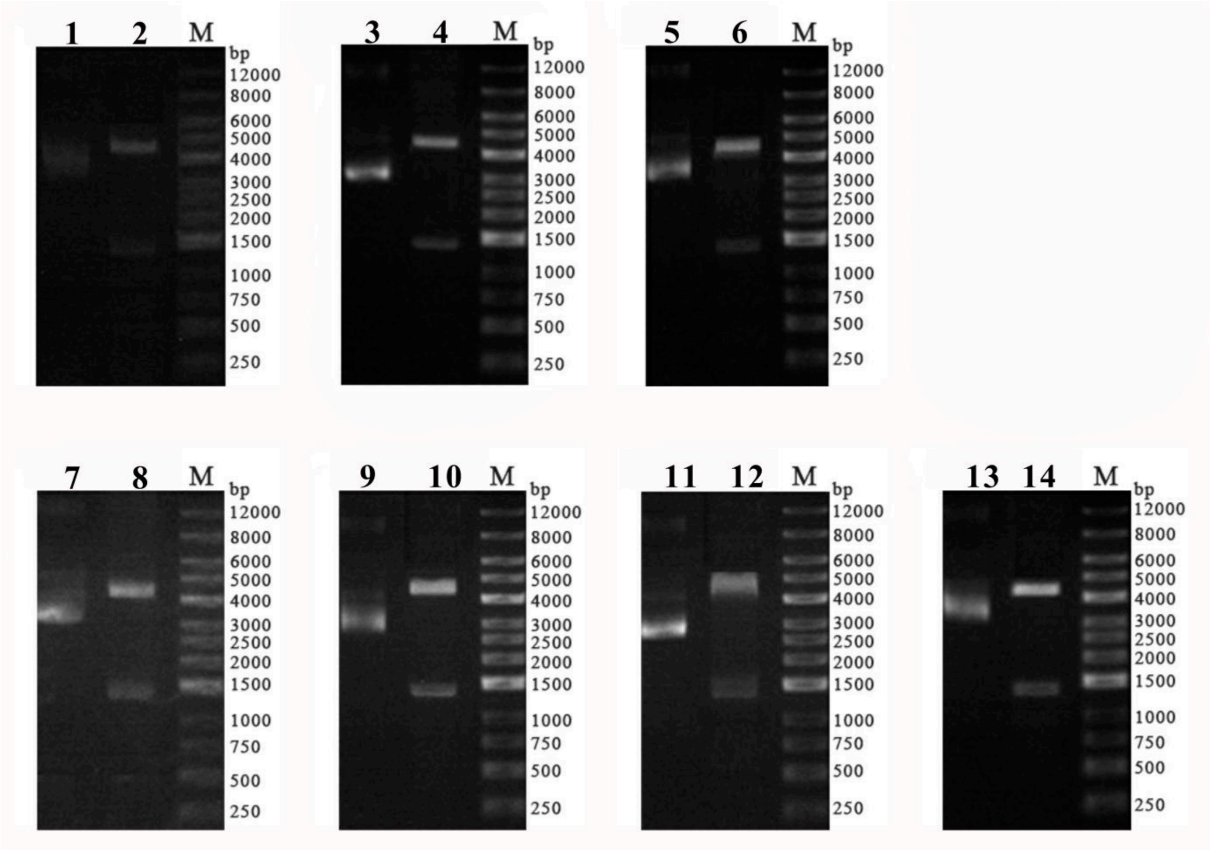

Supplemental Figure S2. Electrophoretic results of double digestion products of 7 nanobody gene prokaryotic expression vectors. 1–2: Pre- and post-digestion products of the pET32a-Nb338 carrier (digestive site: *Apa* I-*Xho* I), 3–4: Pre- and post-digestion products of the pET32a-Nb1712 carrier (digestive site: *Apa* I-*Xho* I), 5–6: Pre- and post-digestion products of the pET32a-Nb799 carrier (digestive site: *Apa* I-*Xho* I), 7–8: Pre- and post-digestion products of the pET32a-Nb1737 carrier (digestive site: *Apa* I-*Xho* I), 9–10: Pre- and post-digestion products of the pET32a-Nb1971 carrier (digestive site: *Apa* I-*Xho* I), 11–12: Pre- and post-digestion products of the pET32a-Nb2000 carrier (digestive site: *Apa* I-*Xho* I), 13–14: Pre- and post-digestion products of the pET32a-Nb2004 carrier (digestive site: *Apa* I-*Xho* I), M: DNA marker.

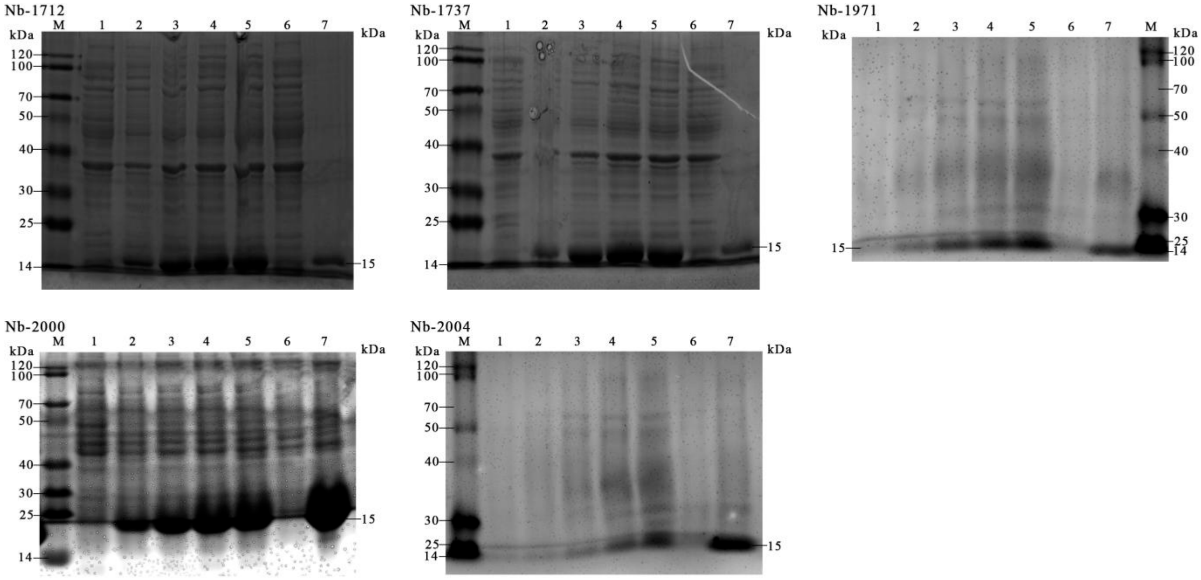

Supplemental Figure S3. Results of induced expression electrophoresis of nanobodies. M: protein marker, 1–5: nanobody proteins were induced for 0, 2, 4, 6, and 8 h, respectively, 6–7: supernatant and precipitation of bacterial liquid after ultrasonic crushing.

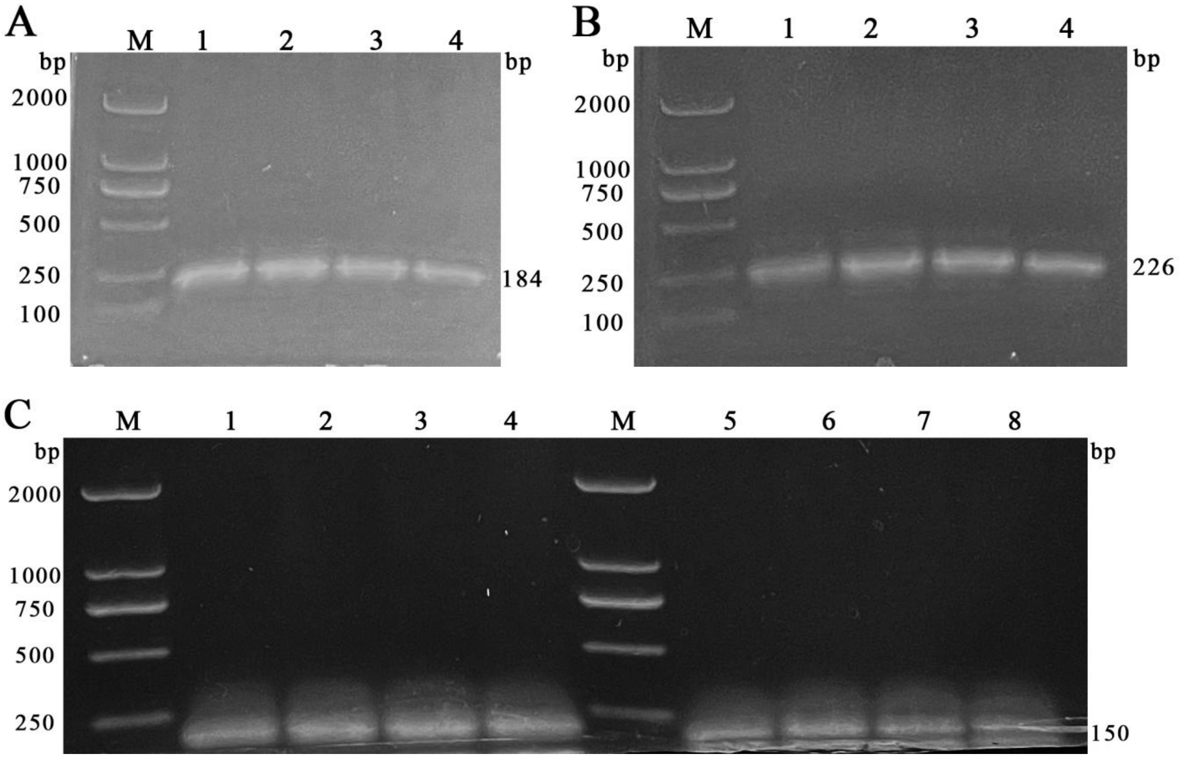

Supplemental Figure S4. A. Result of *INHA* gene PCR product electrophoresis. M: DNA marker, 1-2: *INHA* gene PCR electrophoresis in ovarian tissue, 3-4: *INHA* gene PCR electrophoresis in testicular tissue. B. Result of *Fshb* gene PCR product electrophoresis. M: DNA marker, 1-2: *Fshb* gene PCR electrophoresis in male mouse pituitary, 3-4: *Fshb* gene PCR electrophoresis in female mouse pituitary. C. Result of *GAPDH* gene PCR product electrophoresis. M: DNA marker, 1-2: *GAPDH* gene PCR electrophoresis in ovarian tissue, 3-4: *GAPDH* gene PCR electrophoresis in testicular tissue. 5-6: *GAPDH* gene PCR electrophoresis in male mouse pituitary, 7-8: *GAPDH* gene PCR electrophoresis in female mouse pituitary.
