# Supplementary material for: Screening and Preliminary Identification of Inhibin α Subunit-Specific Nanobodies Through High-Throughput Sequencing Combined with Mass Spectrometry
Source: Animals (Basel). 2026 Jun 25;16(13):1961. doi: 10.3390/ani16131961 (PMC13360298; doi:10.3390/ani16131961)
Supplement: Supplementary file 1 [file animals-16-01961-s001.zip › Supplementary Table.pdf]

Supplementary Table S1. MaxQuant analysis parameter settings

Item	Value
Enzyme	Trypsin
Max Missed Cleavages	2
Precursor Tolerance（Main search）	4.5 ppm
Precursor Tolerance（First search）	20 ppm
MS/MS Tolerance	20 ppm
Fixed modifications	Carbamidomethyl (C)
Variable modifications	Oxidation (M) Acetyl (Protein N-term)
Database	uniprot-Camelus [9836]-52909-20220128.fasta
Database pattern	Target-Reverse
PSM FDR	0.01
Protein FDR	0.01
Site FDR	0.01

Supplementary Table S2. Statistics of Protein Identification Outcomes

Sample	Protein count	Peptide count	PSM identification count
pre-immune serum	165	918	1966
post-immune serum	170	937	1842
Inhibin-α specific antibody	156	357	422
