# Supplementary figures and images for: Screening and Preliminary Identification of Inhibin α Subunit-Specific Nanobodies Through High-Throughput Sequencing Combined with Mass Spectrometry
Source: Animals (Basel). 2026 Jun 25;16(13):1961. doi: 10.3390/ani16131961 (PMC13360298; doi:10.3390/ani16131961)

# OTU1712\_13\_65205

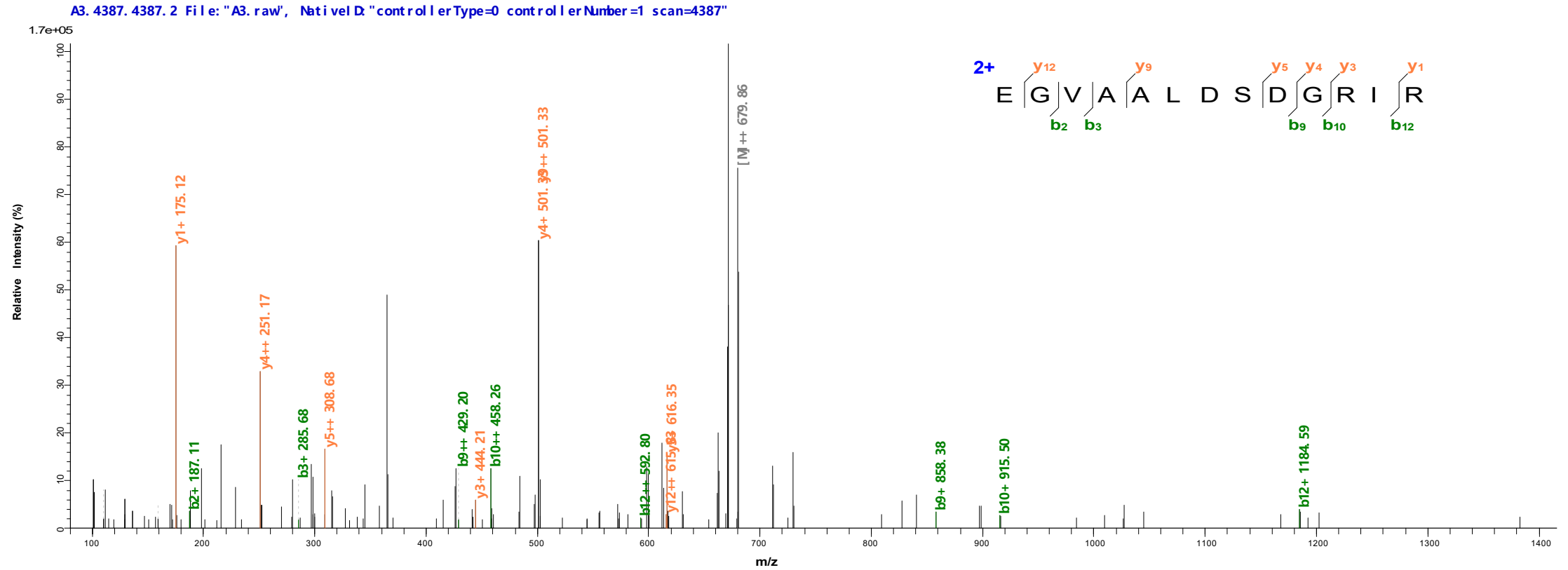

# OTU573\_13\_106697

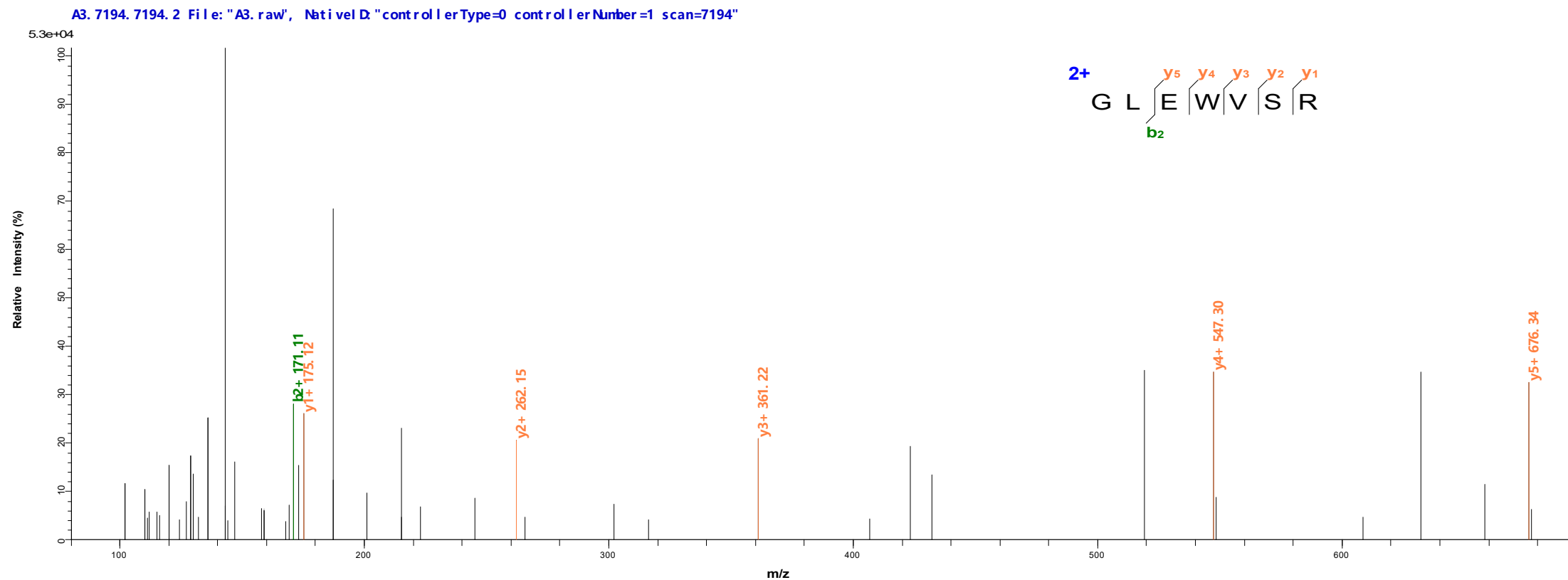

# OTU267\_13\_97272

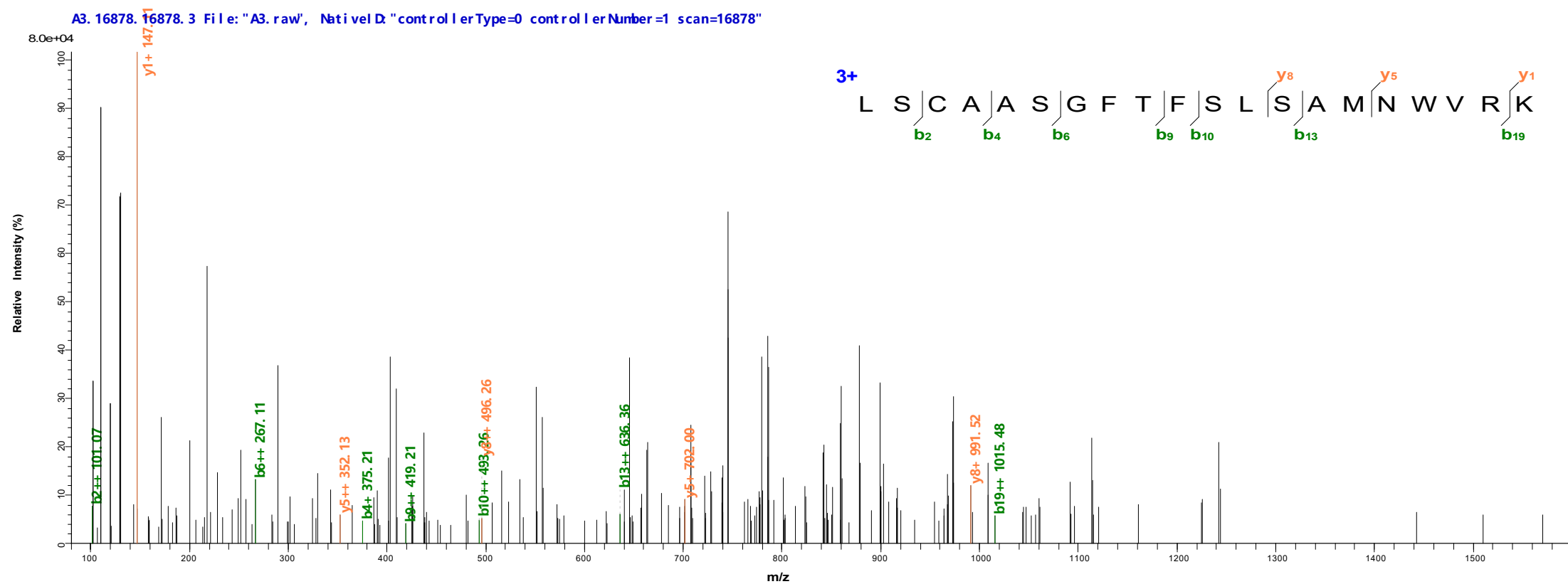

# OTU2000\_13\_90812

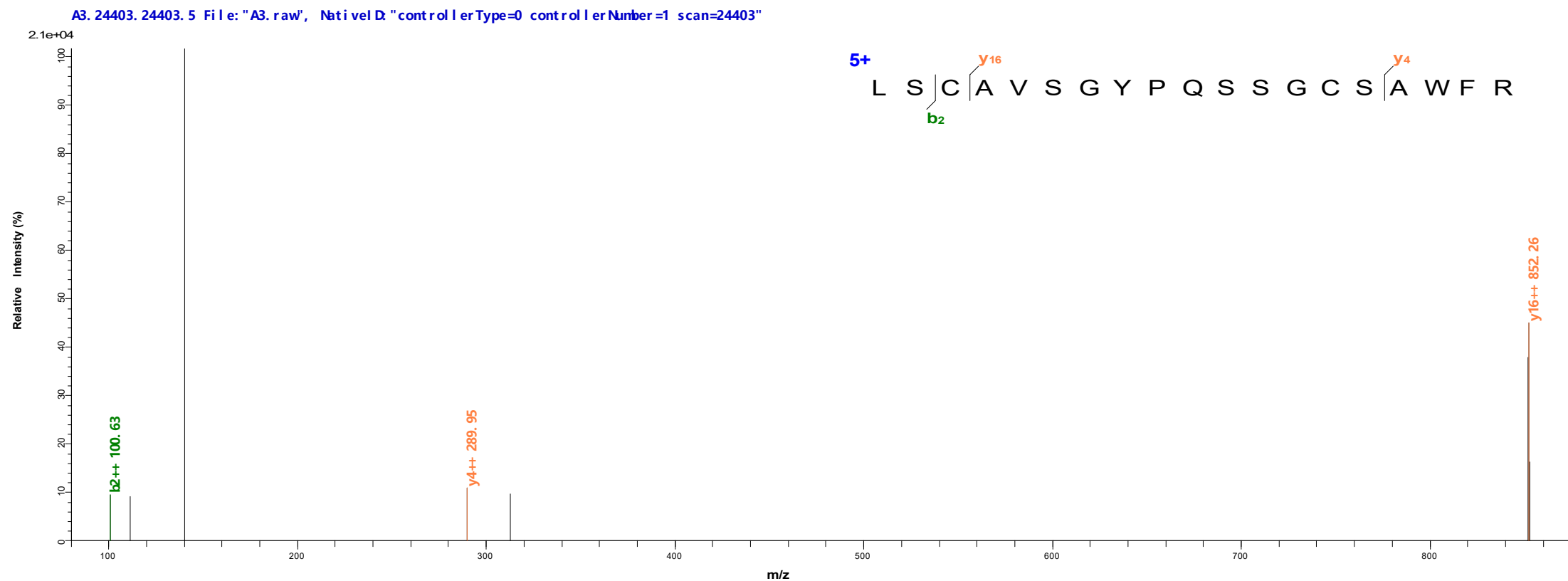

# OTU1971\_13\_100830

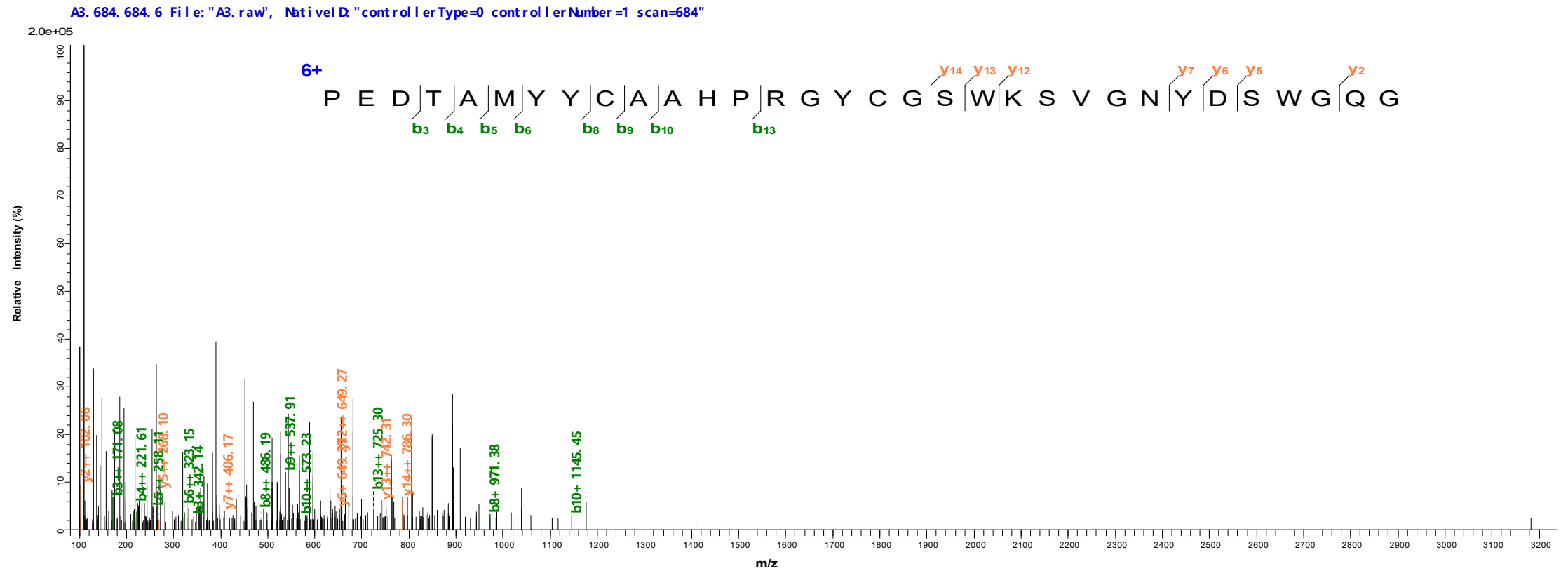

# OTU799\_13\_108715

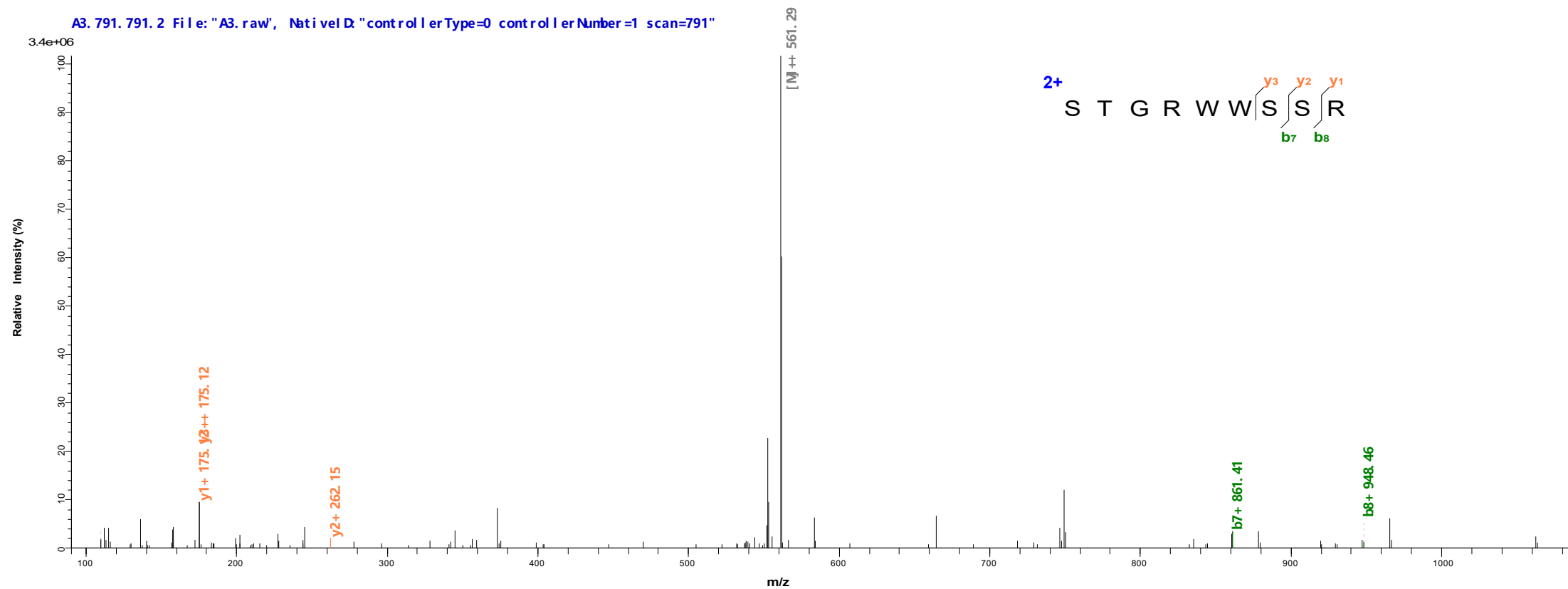

Supplement: Supplementary file 1 [file animals-16-01961-s001.zip › gas phase fragmentation spectra of peptides of inhibin α specific antibodies.pdf]
